# A High‐Loading Zn Single‐Atom Nanozyme Targets the Zn/HIF‐1α/GLUT1 Axis to Disrupt Glucose Metabolic Reprogramming and Remodel the Tumor Immune Microenvironment

**DOI:** 10.1002/advs.76780

**Published:** 2026-07-23

**Authors:** Zhenxin Wang, Yuhang Tang, Ronghao Yue, Jingtao Xu, Yong Tao, Jie Chen, Shipeng Li, Guosheng Zhao, Jinfang Xue, Mingwei Sun, Fenglei Gao, Yunsheng Ou, Yang Wang

**Affiliations:** ^1^ Department of Orthopaedic Surgery The First Affiliated Hospital of Chongqing Medical University Chongqing Medical University Chongqing China; ^2^ Department of Emergency Medicine Center Sichuan Provincial People's Hospital University of Electronic Science and Technology of China Chengdu Sichuan China; ^3^ Department of Core Laboratory Sichuan Provincial People's Hospital University of Electronic Science and Technology of China Chengdu Sichuan China; ^4^ Department of Orthopaedic Surgery The Second Affiliated Hospital of Chongqing Medical University Chongqing Medical University Chongqing China; ^5^ Key Laboratory of New Drug Research and Clinical Pharmacy Xuzhou Medical University Jiangsu China

**Keywords:** disulfidptosis, immunotherapy, pyroptosis, single‐atom nanozyme, TRPML1

## Abstract

Highly metastatic osteosarcoma exhibits marked resistance to conventional therapies, driven by glucose‐metabolic reprogramming together with disturbed zinc homeostasis. This study reveals that the TRPML1/HIF‐1α/GLUT1 axis as a mechanistic link between lysosomal zinc homeostasis and glucose‐metabolic regulation, thereby dictating tumor cell fate. On this basis, we developed a TRPML1‐activation‐based single‐atom nanozyme platform with dual enzymatic activities (ZMG@CS) to synchronously collapse ionic and redox homeostasis within lysosomes. Following the lysosomal accumulation of ZMG@CS, high‐density Zn single‐atom sites enabled peroxidase‐like catalysis, whereas the delivered glucose oxidase (GOx) acidified the local environment through glucose consumption, collectively amplifying oxidative stress. TRPML1 activation promoted Zn^2+^ flux and ROS signaling to inhibit HIF‐1α activity, thereby suppressing GLUT1 and restricting glucose uptake. Consequently, NADPH/GSH regeneration was impaired, causing a collapse of reducing power and triggering disulfidptosis. Simultaneously, zinc overload and ROS cooperatively exacerbated lysosomal membrane damage, activated the NLRP3 inflammasome, and induced caspase‐1‐dependent pyroptosis. These dual death programs synergistically enhanced immunogenic cell death and further reversed the immunosuppressive tumor microenvironment. Together, this study establishes the TRPML1/HIF‐1α/GLUT1 axis as a critical pathway linking lysosomal zinc signaling to glucose‐metabolic vulnerability and provides a new therapeutic avenue for aggressive osteosarcoma.

## Graphical abstract

A high‐loading Zn single‐atom nanozyme (ZMG@CS) delivers ML‐SA5 and GOx to lysosomes. ML‐SA5 activates TRPML1 to release endogenous Zn^2^^+^, while the nanozyme provides exogenous Zn^2^^+^ and GOx‐driven acidification amplifies ROS production. Together, these effects suppress the HIF‐1α/GLUT1 axis, disrupt glucose and redox homeostasis, induce disulfidptosis and pyroptosis, and remodel the osteosarcoma immune microenvironment.

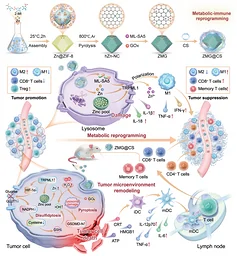

## 1 Introduction

Highly metastatic osteosarcoma (OS) remains a major clinical challenge because metastasis is associated with a sharp decline in 5‐year survival [1, 2]. Compared with localized tumors, it is characterized by two mutually reinforcing metabolic rewiring programs. The first is glucose‐metabolic reprogramming, which drives tumor cells to rely on aerobic glycolysis even under oxygen‐sufficient conditions to meet the energetic demands of rapid proliferation and metastasis [3, 4, 5]. Aberrant overexpression of glucose transporter 1 (GLUT1) is a key hallmark of this phenotype, with elevated levels correlating with increased metastatic risk and contributing to reduced overall survival in patients [6, 7]. The second is dysregulated ionic metabolism, particularly reduced bioavailable zinc in OS, which may relieve zinc‐mediated suppression of hypoxia‐inducible factor‐1α (HIF‐1α) expression and stability within the hypoxic tumor microenvironment (TME) [8, 9]. Accumulation of HIF‐1α can potentiate GLUT1‐mediated glucose uptake and glycolytic flux while upregulating immune‐evasion molecules such as PD‐L1, ultimately establishing a vicious cycle in which ionic imbalance and glycolytic dysregulation reinforce one another [10, 11, 12]. This interplay helps explain why interventions targeting a single metabolic pathway often produce limited and unstable efficacy [13, 14, 15]. Therefore, simultaneous disruption of metabolic reprogramming and ionic homeostatic imbalance may provide a promising strategy for treating metastatic OS.

As a core regulator of hypoxic signaling in the TME, HIF‐1α promotes lactate accumulation by transcriptionally upregulating glycolysis‐associated genes, thereby driving tumor proliferation, metastasis, and therapeutic resistance [16, 17, 18]. Against this background, we propose and validate HIF‐1α as a pivotal hub linking ion homeostasis with glucose‐metabolic reprogramming. Lysosomes are particularly important in this context because they are recognized as major intracellular reservoirs of Zn^2+^ [19, 20, 21]. The internally enriched Zn^2+^ contributes to cellular stress responses and metabolic regulation after release through the transient receptor potential mucolipin 1 (TRPML1) channel on the lysosomal membrane [22, 23, 24]. Activation of TRPML1 dynamically redistributes Zn^2+^ between lysosomes and the cytosol, thereby imposing an intrinsic constraint on HIF‐1α. In this study, exogenous Zn^2+^ supplementation led to a concomitant decrease in HIF‐1α expression and stability. Enhanced lysosomal Zn^2+^ mobilization combined with exogenous Zn^2+^ supplementation suppressed HIF‐1α signaling and concurrently inhibited GLUT1‐mediated glucose uptake. This process was accompanied by the collapse of the intracellular reducing defenses, including impairment of the NADPH/GSH regeneration axis and aberrant disulfide accumulation, which in turn caused actin cytoskeletal disassembly and ultimately triggered disulfidptosis [25, 26, 27]. In parallel, TRPML1‐mediated reactive oxygen species (ROS) production and zinc overload synergistically initiated lysosomal disruption and activated NLRP3 inflammasome‐related pathways, thereby inducing immunogenic pyroptosis and potentially reversing the immunosuppressive microenvironment [28, 29]. Thus, the TRPML1/HIF‐1α/GLUT1 axis links ion homeostasis to glucose‐metabolic signaling and provides mechanistic support for anti‐tumor strategies that exploit metabolic vulnerabilities.

However, within the complex TME, TRPML1 activation alone often fails to achieve sustained and robust metabolic suppression. Limited endogenous zinc availability and insufficient ROS signaling further constrain prolonged activation and amplification of the Zn‐HIF‐1α axis [30]. In addition, TRPML1 agonists commonly suffer from poor solubility and limited stability in systemic circulation, reducing their effective exposure at tumor sites [31, 32]. Moreover, strategies that rely solely on nonspecific oxidative stress may increase off‐target damage and lead to variable therapeutic outcomes [33, 34]. Therefore, a localized approach capable of simultaneously providing Zn^2+^, suppressing tumor metabolism, and amplifying oxidative stress is needed to overcome the adaptive survival capacity of highly metastatic OS.

To this end, we designed a high‐loading Zn single‐atom nanozyme as an exogenous Zn donor and developed a TRPML1‐activation‐based metabolic sensitizer (Scheme 1). The Zn single‐atom nanozyme served as a self‐catalytic drug carrier to co‐load the TRPML1 agonist (ML‐SA5) and glucose oxidase (GOx), and was further coated with chondroitin sulfate (CS) to obtain CD44‐targeted nanoparticles (ZMG@CS) that preferentially recognize OS cells with high CD44 expression. Compared with previously reported nanozymes, which typically suffer from low metal‐atom loading, our system achieved a relatively high Zn single‐atom loading (5.93 wt%). The dense Zn‐N_4_ coordination, coupled with high defect density, enabled Zn single atoms to function as catalytic centers with pronounced peroxidase‐like (POD‐like) activity [35, 36]. Following lysosomal enrichment, this structure facilitates localized amplification of zinc supply and ROS generation. More importantly, precise TRPML1 activation by ML‐SA5 converted combined endogenous and exogenous zinc signals into sustained suppression of the HIF‐1α/GLUT1 pathway. In concert with GOx, this produced a dual blockade of glucose metabolism by limiting glucose uptake and accelerating glucose consumption, thereby strongly suppressing NADPH/GSH regeneration and ultimately inducing disulfidptosis. In parallel, lysosomal zinc homeostasis disruption and oxidative stress cooperatively increased lysosomal membrane damage, triggering subsequent pyroptotic responses. Notably, pyroptosis triggers the rapid release of tumor‐associated antigens and damage‐associated molecular patterns (DAMPs), thereby efficiently recruiting immune cells and activating anti‐tumor immune responses [37, 38]. Collectively, this study shows that TRPML1 activation triggers ion‐homeostasis‐imbalance‐driven glucose‐metabolic reprogramming, leading to metabolic suppression and organelle‐targeted therapy against highly metastatic OS and providing a new approach for exploiting metabolic vulnerabilities in OS.

**SCHEME 1:**
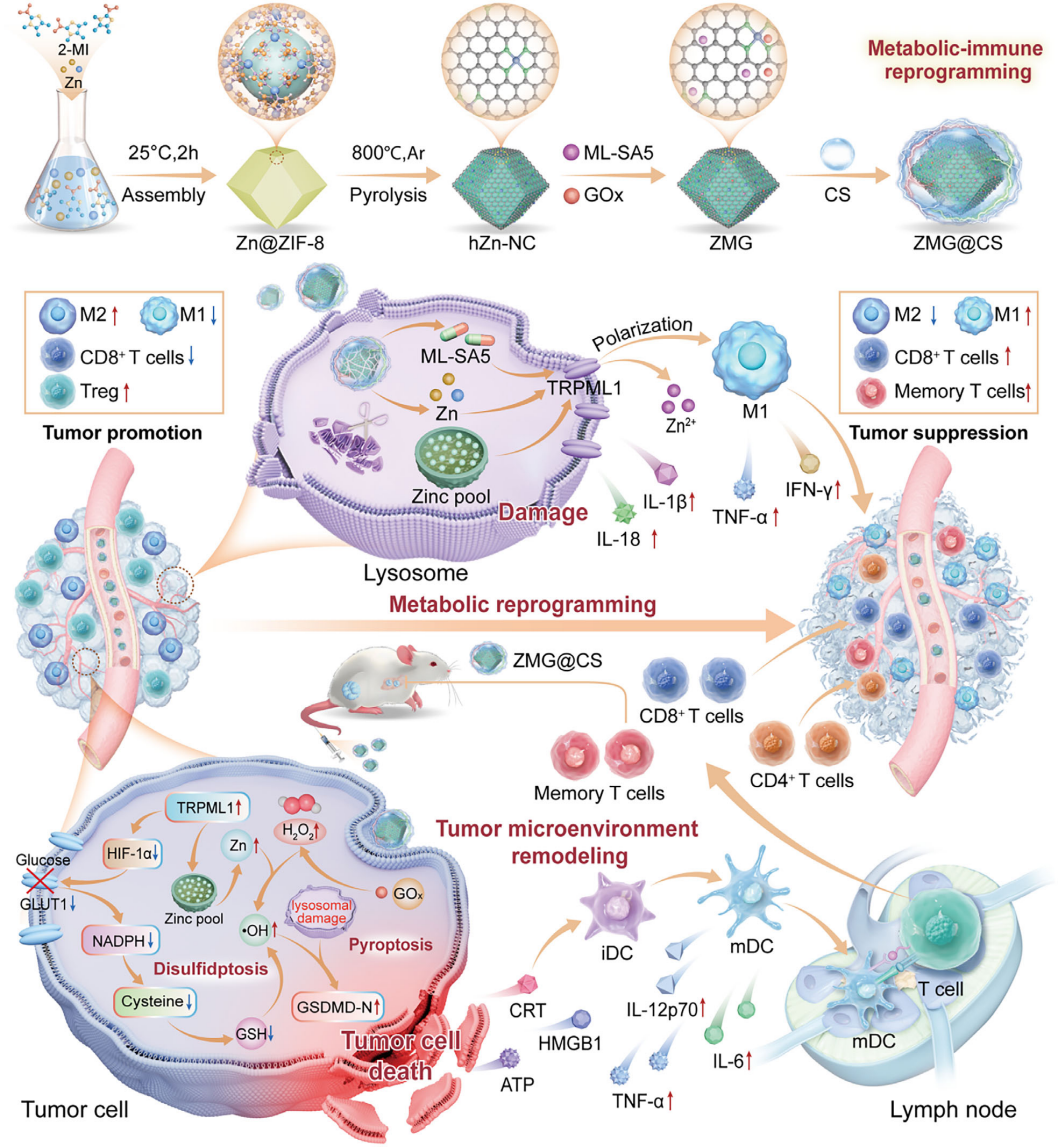
Schematic illustration of the synthesis and therapeutic mechanism of the ZMG@CS nanoplatform for metabolic‐immune reprogramming in highly metastatic OS.

## 2 Results and Discussion

### 2.1 Zinc Homeostasis Remodeling Suppresses the HIF‐1α/GLUT1 Axis

Single‐sample Gene Set Enrichment Analysis (ssGSEA) demonstrated a notable increase in the zinc ion‐responsive signature in tumor tissues relative to normal tissues (Figure 1a), suggesting enhanced zinc‐related stress programs in the TME. Consistent with this finding, pharmacological activation of TRPML1 with ML‐SA5 decreased the viability of 143B cells in a dose‐dependent manner (Figure 1b). Reduced red fluorescence in acridine orange (AO) staining indicated increased lysosomal membrane permeability and lysosomal zinc release (Figure 1c). Notably, quantification of cellular zinc content demonstrated that ML‐SA5 did not markedly elevate total intracellular zinc relative to the control, whereas ZnCl_2_ caused a pronounced increase (Figure 1d). These findings imply that TRPML1 activation primarily influences the dynamic redistribution of intracellular zinc, whereas exogenous ZnCl_2_ directly raises the total cellular zinc level.

**FIGURE 1:**
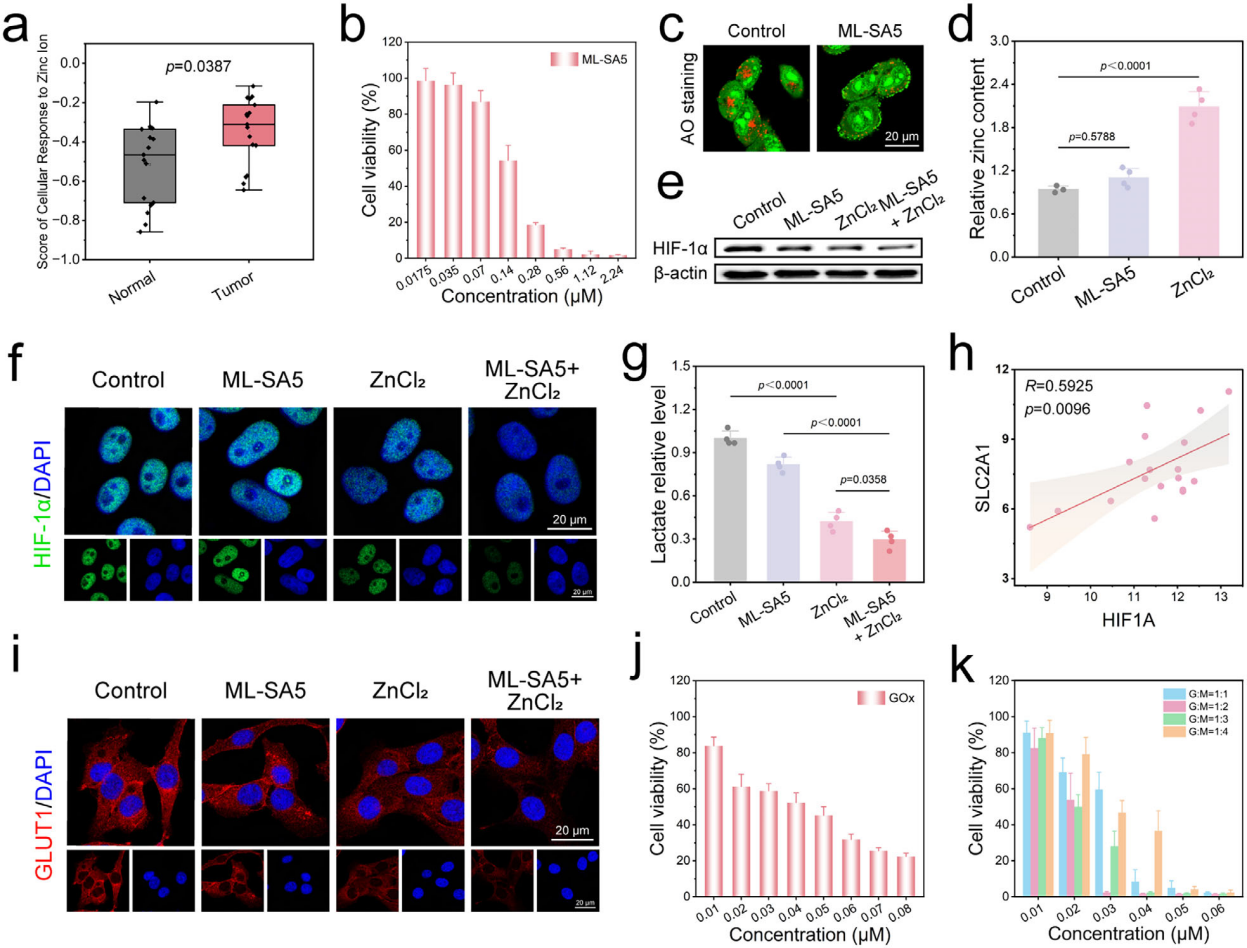
(a) ssGSEA‐based scoring of the zinc ion‐responsive signature in normal versus tumor tissues (n = 17). (b) Cell viability of 143B cells following treatment with ML‐SA5 at different concentrations (n = 4). c) AO staining of cells after ML‐SA5 treatment. (d) Relative intracellular Zn^2^^+^ levels in cells treated with ML‐SA5 or ZnCl_2_ (n = 4). (e) WB analysis of HIF‐1α expression in cells under various treatments. (f) Immunofluorescence staining of HIF‐1α in cells under different conditions, (g) together with measurement of lactate levels (n = 4). (h) Bioinformatic correlation analysis between HIF1A and SLC2A1 expression. (i) Immunofluorescence staining of GLUT1 in cells from the indicated treatment groups. (j) Cell viability of 143B cells treated with GOx at the indicated concentrations (n = 5). (k) Changes in cell viability with varying drug concentrations under different ratios of GOx and ML‐SA5 (n = 5). Statistical analyses were performed using one‐way ANOVA followed by Tukey's multiple‐comparisons test. Significance: ns, not significant; ^*^ *p* < 0.05; ^**^ *p* < 0.01; ^***^ *p* < 0.001; ^****^ *p* < 0.0001.

Importantly, enhanced zinc availability coincided with attenuation of the hypoxia‐glucose transport program. Western blot (WB) analysis revealed decreased HIF‐1α expression after combined ZnCl_2_ and ML‐SA5 treatment (Figure 1e), which was further supported by HIF‐1α immunofluorescence staining (Figure 1f). Functionally, intracellular lactate levels were reduced after ZnCl2 exposure and were further decreased by the combined treatment (Figure 1g). Concurrently, bioinformatics analysis indicated a positive correlation between HIF1A and SLC2A1 (the gene encoding GLUT1) expression (Figure 1h). Correspondingly, GLUT1 immunofluorescence intensity was attenuated in the combination group relative to controls (Figure 1i). To further reduce intracellular glucose availability, we examined the cytotoxicity of GOx in 143B cells and observed a clear dose‐dependent effect (Figure 1j). We next optimized the GOx/ML‐SA5 combination by CCK‐8 analysis across a range of ratios and determined that a 1:2 ratio produced the sharpest concentration‐dependent loss of viability, consistent with strong synergy between glucose deprivation and TRPML1 activation (Figure 1k). Collectively, these results support a coherent model in which elevated zinc levels correlate with downregulation of HIF‐1α and its downstream target GLUT1, thereby linking zinc homeostasis to glucose‐metabolic reprogramming in cells.

### 2.2 Design and Characterization of ZMG@CS

To establish a self‐reinforcing nanosystem capable of amplifying metabolic suppression, a single‐atom nanoenzyme was first synthesized via high‐temperature pyrolysis. This nanoenzyme features isolated zinc atom sites anchored within a nitrogen‐coordinated carbon‐based framework, thereby serving as a high‐density zinc reservoir while providing POD‐like enzyme activity. Pyrolysis of ZIF‐8 under an argon atmosphere yielded a porous nanostructure (denoted as hZn‐NC) with high zinc loading, whose morphology was consistent with a metal‐organic framework‐derived carbonized scaffold (Figure 2a). Inductively coupled plasma optical emission spectroscopy (ICP‐OES) revealed an average Zn content of 5.93 wt%, significantly higher than typical Zn doping levels reported in the literature (Table S1). Increasing the initial mass of ZIF‐8 and enhancing the internal Zn source during pyrolysis effectively suppressed Zn loss. Energy‐dispersive X‐ray spectroscopy (EDS) mapping confirmed the uniform distribution of Zn, C, and N signals throughout the structure (Figure 2b). High‐angle annular dark‐field scanning transmission electron microscopy (HAADF‐STEM) further revealed atomic‐scale bright spots corresponding to Zn single atoms (Figure 2c). A three‐dimensional Gaussian fitting analysis of atomic‐intensity overlap was applied to reconstruct the selected region in Figure 2c, where isolated Zn atoms were clearly visualized within the hZn‐NC matrix. X‐ray diffraction (XRD) indicated that the precursor underwent a phase transition toward an amorphous‐carbon‐dominated structure, with no detectable crystalline Zn species, which was consistent with atomic dispersion rather than cluster formation (Figure 2d). N_2_ adsorption isotherms indicated that hZn‐NC possessed a substantial specific surface area of 1161.24 m^2^/g and a hierarchical porous structure, providing a structural basis for enzyme and small‐molecule loading (Figure 2e). Based on the standard curves for ML‐SA5 and GOx, their loading contents were quantified by UV–vis spectrophotometry as 2.39% and 12.88%, respectively, validating the successful construction of ZMG@CS (Figures S1 and S2). Dynamic light scattering (DLS) revealed a narrow hydrodynamic size distribution with a polydispersity index of 0.099, signifying excellent colloidal stability (Figure 2f). After chondroitin sulfate modification, the zeta potential decreased to −20.37 mV, supporting successful surface engineering and acquisition of tumor‐targeting capability (Figure 2g).

**FIGURE 2:**
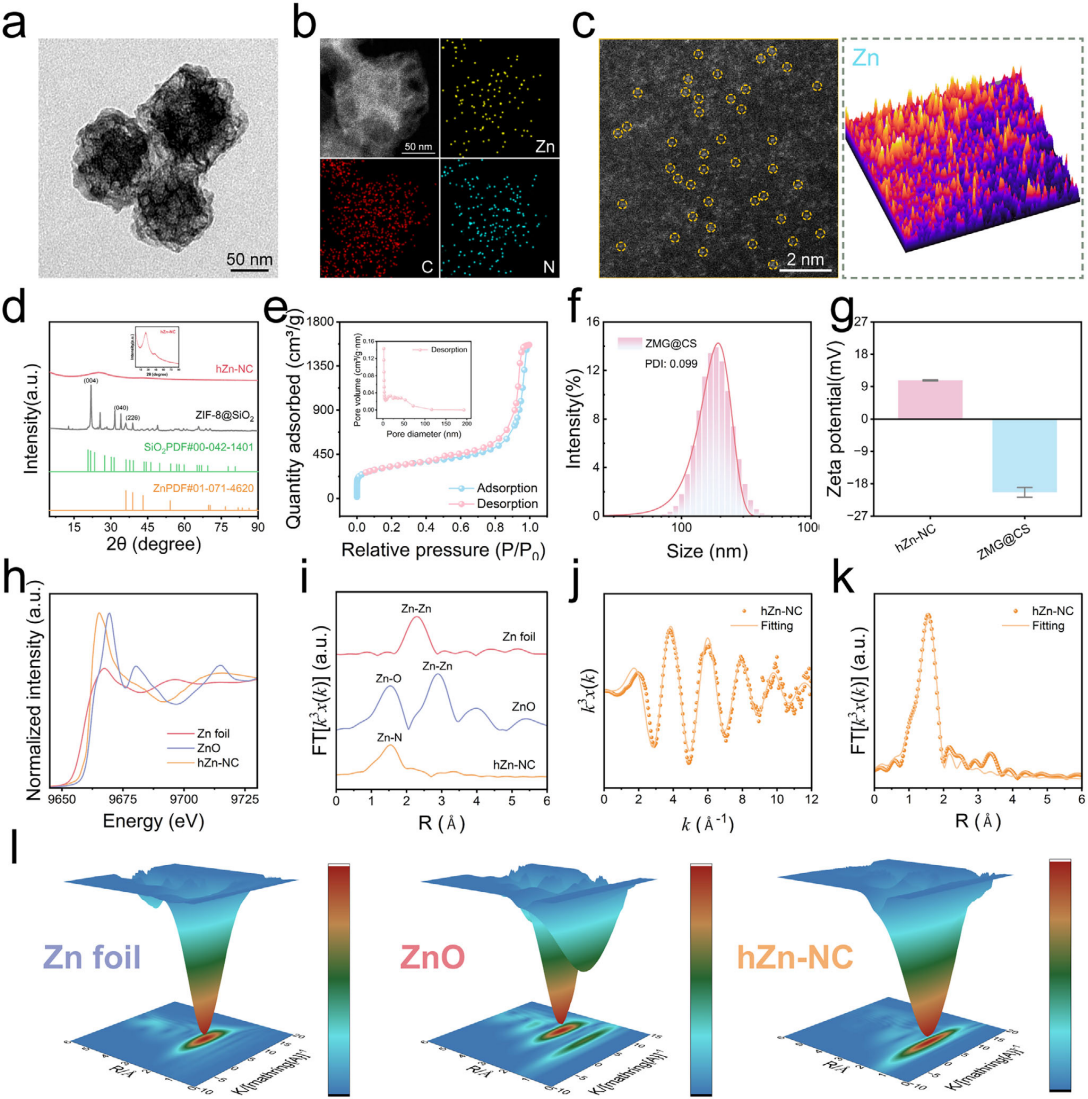
(a) TEM image of ZMG@CS. b) HAADF‐STEM image of ZMG@CS and corresponding STEM‐EDS elemental maps of Zn, C, and N. (c) Aberration‐corrected STEM image of ZMG@CS with highlighted Zn features and the corresponding Zn signal‐intensity rendering. (d) XRD patterns of hZn‐NC with ZIF‐8@SiO_2_ and standard cards. (e) N_2_ adsorption–desorption isotherms of hZn‐NC measured at 77 K; inset: pore‐size distribution derived from the adsorption branch. (f) DLS size distribution profiles of ZMG@CS with the PDI. (g) Zeta‐potential measurements of hZn‐NC and ZMG@CS. (h) Zn K‐edge XANES spectra of hZn‐NC, Zn foil, and ZnO. (i) Fourier‐transformed EXAFS spectra in R space. EXAFS fitting of hZn‐NC in j) *k* space and (k) R space. (l) WT EXAFS plots of Zn foil, ZnO, and hZn‐NC.

X‐ray absorption fine structure (XAFS) analysis confirmed the chemical state and coordination environment of Zn in ZMG@CS, providing conclusive evidence of single‐atom coordination. The Zn K‐edge X‐ray absorption near‐edge structure (XANES) spectrum, located between those of Zn foil and ZnO, indicated a partial positive charge on the Zn atoms (Figure 2h). Fourier‐transformed *k* ^3^‐weighted extended X‐ray absorption fine structure (EXAFS) revealed the absence of Zn‐Zn scattering peaks, pointing to isolated sites dominated by Zn─N coordination (Figure 2i). The best‐fitting results are shown in Figure 2j,k and Figure S3 and Table S2, indicating a Zn─N bond length of 2.04 Å and a coordination number of approximately 4.08. The relatively high coordination number may result from high N content that facilitates axial adsorption of N species on a subset of Zn sites. The Zn K‐edge oscillations observed in the wavelet transform (WT) further confirmed the absence of metallic Zn and ZnO, together with HAADF‐STEM supported an atomically dispersed Zn state (Figure 2l). Collectively, these results indicate that zinc is embedded as isolated sites within the porous framework, achieving high site density while retaining stimulus‐responsive Zn availability.

### 2.3 Zn Coordination Chemistry and Associated Catalytic Properties of ZMG@CS

The X‐ray photoelectron spectroscopy (XPS) survey spectrum clearly displayed distinct C, N, O, and Zn signals in ZMG@CS, confirming the successful integration of Zn into the carbon‐based framework (Figure 3a). The high‐resolution Zn 2p spectrum further showed characteristic peaks at approximately 1021.8 and 1044.9 eV, corresponding to Zn 2p_3/2_ and Zn 2p_1/2_, respectively. No characteristic signals associated with metallic Zn or ZnO clusters were observed, indicating that Zn primarily exists in a highly dispersed atomic state (Figure 3b). The C 1s spectrum showed that the C─C bonds at 284.8 eV accounted for 54.94%, indicating a continuous carbon framework that supports stable electronic transport. The C─N/C─O peak at 286.37 eV (32.54%) indicated abundant polar bonds and N‐doped structures within the carbon framework, providing high‐density anchoring sites for Zn atoms. The remaining C═N or C═O group signals suggested a defect‐enriched carbon framework, which is beneficial for local electronic structure modulation and promotes POD‐like catalytic processes (Figure 3c,d). Further high‐resolution N 1s spectra revealed four distinct N species: pyridinic‐N (53.37%), M‐N (24.87%), pyrrolic‐N (16.04%), and graphitic‐N (5.71%). The predominance of pyridinic‐N (>50%) indicates that coordination‐type N species provide a key coordination environment for stabilizing single‐atom sites. M‐N served as strong evidence of direct Zn─N coordination, whereas the remaining two N species optimized electron transfer and reaction kinetics while maintaining structural stability (Figure 3e,f). Collectively, the peak distribution and quantitative ratios of the C 1s and N 1s signals point to a defect‐enriched N‐doped carbon framework with pronounced metal‐nitrogen coordination, providing a structural rationale for high catalytic activity and single‐atom site stability.

**FIGURE 3:**
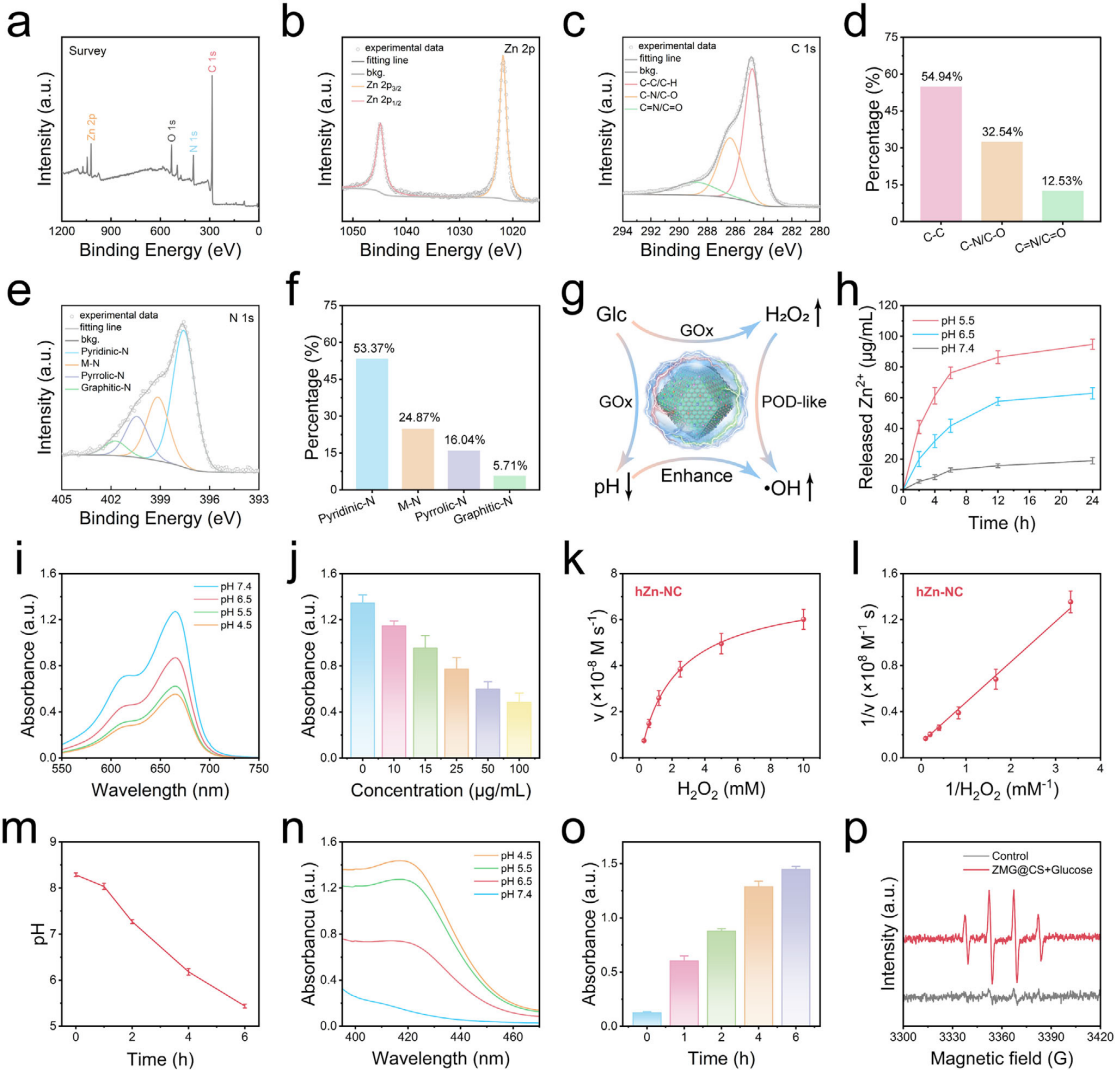
(a) XPS survey spectrum of ZMG@CS. (b) High‐resolution Zn 2p XPS spectrum of ZMG@CS with peak deconvolution. (c) High‐resolution C 1s XPS spectrum of ZMG@CS. (d) Relative percentages of the deconvoluted C 1s components. (e) High‐resolution N 1s XPS spectrum of ZMG@CS. (f) Relative percentages of the deconvoluted N 1s components. (g) Schematic illustration of the ZMG@CS‐enabled catalytic cascade. (h) Time‐dependent Zn^2+^ release assay of ZMG@CS in buffers with different pH values (n = 3). (i) UV–vis spectra acquired to monitor the MB oxidation reaction catalyzed by ZMG@CS at different pH. (j) Concentration‐dependent MB consumption by ZMG@CS. (k) Steady‐state kinetic measurements using TMB as the chromogenic substrate by varying H_2_O_2_ concentrations. (l) Lineweaver‐Burk (double‐reciprocal) plot derived from the TMB‐based steady‐state kinetic data (n = 3). (m) Time‐dependent pH monitoring of a glucose‐containing system in the presence of ZMG@CS (n = 3). UV–vis spectra of the ABTS system recorded under (n) different pH values and (o) different reaction times to monitor H_2_O_2_ generation in the presence of ZMG@CS (n = 3). (p) ESR spin‐trapping measurements for ROS detection in the ZMG@CS + glucose system.

The therapeutic rationale of this study involves coupling glucose oxidation with POD‐like enzyme catalysis to amplify redox stress while enabling Zn release under acidic conditions (Figure 3g). Given the high acidity of lysosomes, Zn release was evaluated at different pH values. ZMG@CS exhibited substantially enhanced Zn release under acidic conditions, whereas release was markedly reduced in neutral environments (Figure 3h). This acid‐responsive behavior supports preferential Zn overload within acidic intracellular compartments rather than in systemic circulation, which is expected to improve tumor selectivity. POD‐like catalytic performance was characterized by methylene blue (MB) oxidation. The extent of MB oxidation increased with both acidity and ZMG@CS concentration, indicating higher activity in lysosome‐like environments (Figure 3i,j). Enzyme kinetics were determined using the TMB chromogenic reaction. Rate curves obtained by varying hydrogen peroxide (H_2_O_2_) concentrations and the corresponding double‐reciprocal plots supported enzyme‐like kinetic behavior (Figure 3k,l). These data demonstrate that the Zn single‐atom framework functions as an efficient POD‐like catalyst under TME‐relevant conditions. Incorporation of GOx within ZMG@CS enables glucose conversion to gluconic acid and H_2_O_2_ generation, thereby lowering the local pH and providing a substrate for POD‐like catalysis [39]. In glucose containing systems, the pH decreased over time after ZMG@CS addition (Figure 3m). H_2_O_2_ generation was monitored via ABTS oxidation, with UV–vis spectra revealing enhanced signals at lower pH and extended reaction times (Figure 3n,o). Electron spin resonance (ESR) further confirmed the formation of hydroxyl radicals (•OH) with characteristic patterns in the presence of glucose (Figure 3p), demonstrating that this cascade exploits endogenous metabolic substrates to generate oxidative stress. In summary, this system forms a closed‐loop amplification cascade driven by glucose consumption, which self‐sustains acidification and H_2_O_2_ supply. This chemical foundation primes subsequent lysosomal ionic stress and glucose‐metabolic suppression.

### 2.4 Lysosomal Zinc Overload and Metabolic Collapse Consistent with Disulfidptosis

Efficient lysosomal delivery and coordinated disruption of cellular homeostasis are prerequisites for inducing cell death [40, 41]. To assess whether CS modification could facilitate cellular internalization, FITC‐labeled ZMG@CS was first incubated with L929 normal mouse fibroblasts with low CD44 expression and 143B osteosarcoma cells with high CD44 expression. Under the same incubation conditions, the fluorescence signal of ZMG@CS‐FITC in L929 cells was markedly weaker than that observed in osteosarcoma cells (Figure 4a; Figure S4a,b). In addition, FITC‐labeled ZMG and ZMG@CS were separately incubated with 143B and K7M2 cells. The intracellular FITC signal increased with prolonged incubation, and ZMG@CS‐FITC showed greater cellular uptake than ZMG‐FITC (Figure S4c–g). We next evaluated biocompatibility and tumor selectivity. In both OS cell lines, hZn‐NC exhibited marked dose‐dependent cytotoxicity (Figure 4b). ZMG@CS induced dose‐dependent cytotoxicity in OS cells, whereas hFOB 1.19 cells maintained relatively high viability under the same conditions. Compared with ZMG@CS, ZMG showed reduced cytotoxicity toward osteosarcoma cells but slightly increased cytotoxicity toward hFOB 1.19 cells. These results suggest that the CS coating may enhance the preferential activity of the nanoplatform against CD44‐expressing OS cells without markedly increasing nonspecific toxicity toward normal osteoblasts (Figure 4c; Figure S5). Among different treatment groups, the intact cascade‐configured ZMG@CS demonstrated the strongest inhibition of cellular activity, significantly outperforming partially assembled component systems and further indicating synergistic interactions between the carrier and both drugs (Figure 4d).

**FIGURE 4:**
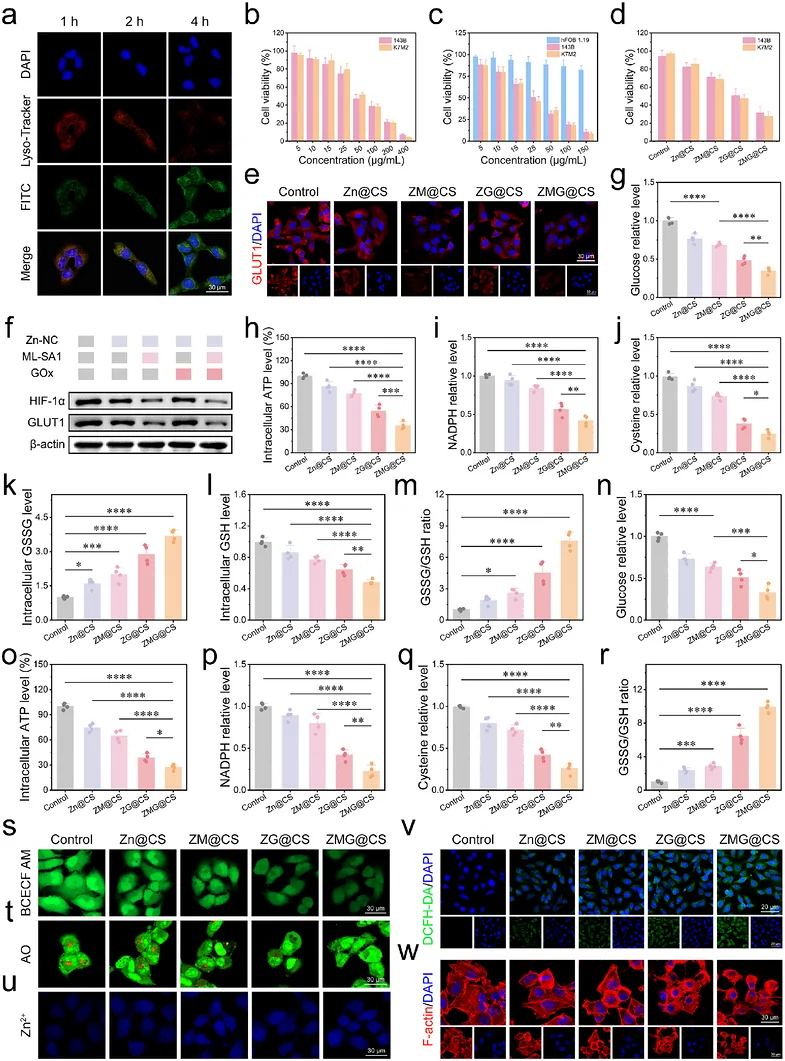
(a) Confocal imaging of FITC‐labeled ZMG@CS with Lyso‐Tracker at different incubation times to evaluate intracellular localization. (b) Cell viability assay of 143B cells treated with Zn@CS at graded concentrations (n = 5). (c) Cell viability assays in different cell lines treated with graded concentrations of ZMG@CS (n = 5). (d) Cell viability assay under different treatment groups (n = 4). (e) Immunofluorescence staining of GLUT1 after the indicated treatments. (f) WB analysis of HIF‐1α and GLUT1 under different treatments. (g) Relative glucose quantification in 143B cells under different conditions (n = 4). (h) Intracellular ATP determination among different experimental groups (n = 4). (i) Assessment of relative NADPH levels in the intervention groups (n = 4). (j) Cysteine level evaluation following the respective group treatments (n = 4). Measurement of (k) intracellular GSSG and l) GSH levels under different conditions (n = 4). (m) Calculation and statistical analysis of the GSSG/GSH ratio. Parallel assays in K7M2 cells across the control and treatment groups for (n) glucose, (o) ATP, (p) NADPH, (q) cysteine, and r) the GSSG/GSH ratio (n = 4). (s) BCECF‐AM fluorescent probe imaging to evaluate intracellular acidity in the indicated groups. (t) AO staining‐based imaging for lysosome‐associated characterization in different groups. (u) Fluorescent imaging with Zinquin ethyl ester to assess intracellular Zn^2+^ following the respective group interventions. (v) Confocal imaging with DCFH‐DA to monitor intracellular ROS, compared among different groups. (w) Fluorescent staining of F‐actin was employed to visualize the cytoskeletal structure in different groups. Statistical analyses were performed using one‐way ANOVA followed by Tukey's multiple‐comparisons test. Significance: ns, not significant; ^*^ *p* < 0.05; ^**^ *p* < 0.01; ^***^ *p* < 0.001; ^****^ *p* < 0.0001.

Given the central role of glucose metabolism in this mechanism, GLUT1 expression and intracellular glucose levels were examined. Immunofluorescence revealed significantly reduced GLUT1 signaling following ZMG@CS treatment (Figure 4e). WB further confirmed regulated changes in HIF‐1α and GLUT1 expression under different drug combinations. HIF‐1α was significantly inhibited following ZMG@CS treatment, supporting TRPML1 activation and subsequent suppression of HIF‐1α/GLUT1 axis (Figure 4f). Functionally, intracellular glucose levels in 143B cells decreased markedly after ZMG@CS treatment, consistent with GOx‐mediated depletion and restricted glucose transport (Figure 4g). Decreased ATP and NADPH levels indicated concurrent impairment of energy production and reductive capacity (Figure 4h,i). Given the sensitivity of disulfidptosis to reductive capacity, intracellular cysteine and glutathione (GSH) pools were further measured. Cysteine levels significantly decreased following ZMG@CS treatment (Figure 4j). Compared with other groups, the ZMG@CS group exhibited the highest oxidized glutathione (GSSG) content and lowest reduced GSH levels, with a significantly elevated GSSG/GSH ratio, reflecting severe disruption of intracellular metabolism and redox homeostasis (Figure 4k–m). In K7M2 cells, parallel alterations in glucose‐metabolic suppression and depletion of reducing equivalents were also observed (Figure 4n–r; Figure S6). To further verify disulfidptosis, non‐reducing SDS‐PAGE western blot analyses of actin were performed. Under non‐reducing conditions, actin in ZMG@CS‐treated cells exhibited pronounced oligomerization with retarded migration and high‐molecular‐weight smear bands, indicating disulfide bond‐mediated actin cross‐linking (Figure S7). These findings are aligned with reported hallmarks of disulfidptosis.

Consistent with GOx‐driven intracellular acidification, the BCECF‐AM probe indicated a substantial increase in intracellular acidity following treatment with GOx‐containing nanoparticles (Figure 4s). AO staining revealed progressive loss of intracellular red fluorescence, suggesting increased lysosomal membrane permeability (Figure 4t). A Zn^2+^ sensitive fluorescent probe detected elevated intracellular Zn signals, supporting Zn delivery and overload (Figure 4u). DCFH‐DA fluorescence imaging revealed markedly elevated intracellular ROS in the ZMG@CS group as a result of multistep cascade catalysis (Figure 4v). Further analysis of cytoskeletal morphology showed clear differences between the PBS and ZMG@CS groups. In contrast to the well‐spread cytoskeleton observed in the PBS group, ZMG@CS‐treated 143B cells exhibited cytoskeletal contraction and collapse (Figure 4w). Together, these findings constitute a characteristic profile consistent with disulfidptosis, a process initiated by TRPML1 activation and subsequent suppression of TRPML1/HIF‐1α/GLUT1 axis and further amplified by intracellular Zn^2+^ overload.

### 2.5 ZMG@CS‐Mediated Immunogenic Pyroptosis

To systematically elucidate ZMG@CS‐induced inflammasome‐associated pyroptosis and immune activation, multiple complementary assays were performed in highly metastatic OS cells. Representative inverted microscopy images revealed that, compared with other treatments, ZMG@CS‐treated cells exhibited marked swelling accompanied by membrane rupture and vacuolization, displaying typical pyroptosis‐associated morphology (Figure 5a). Notably, JC‐1 staining results further demonstrated that 143B cells treated with ZMG@CS exhibited the strongest green fluorescence and the weakest red fluorescence compared to PBS‐ and Zn@CS‐treated cells, indicating severe mitochondrial damage induced by redox imbalance (Figure 5b). Subsequently, we investigated the potential pathways mediating ZMG@CS‐induced pyroptosis. WB analysis showed significant upregulation of NLRP3 following ZMG@CS treatment, accompanied by enhanced caspase‐1 cleavage and marked upregulation of the N‐terminal fragment of gasdermin D (GSDMD), indicating activation of the classical pyroptotic pathway (Figure 5c,d). Notably, increased lactate dehydrogenase (LDH) release supported disruption of cell membrane permeability and further confirmed pore formation (Figure 5e). Because pyroptosis‐mediated pore formation and subsequent lysis cause extensive release of cytoplasmic contents and inflammatory cytokines, we next analyzed inflammatory cytokine secretion by ELISA. Therefore, we further analyzed the secretion of various intracellular inflammatory markers via ELISA assays. The ZMG@CS group showed 14.9‐fold and 6.9‐fold increases in interleukin‐1β (IL‐1β) and interleukin‐18 (IL‐18) levels in the supernatant, respectively, corroborating pyroptosis‐associated inflammasome activation (Figure 5f,g).

**FIGURE 5:**
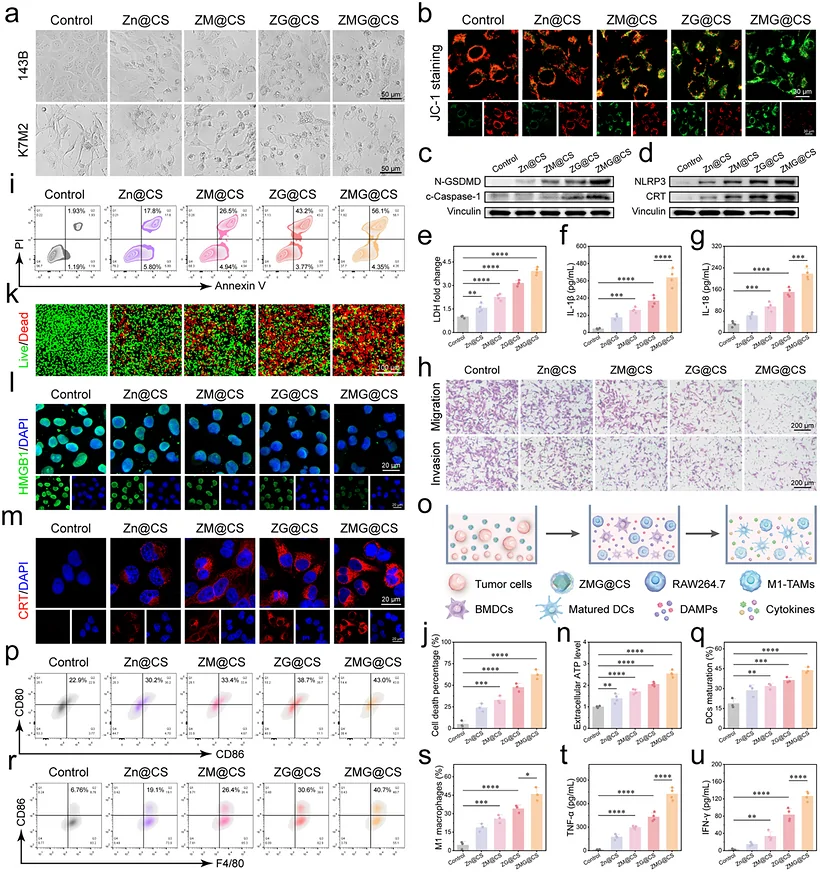
(a) Representative bright‐field micrographs of 143B and K7M2 cells after various treatments. (b) JC‐1 fluorescence staining for assessing mitochondrial membrane potential (MMP) in different groups. (c) WB analysis of pyroptosis‐associated proteins with vinculin as a loading control. (d) WB analysis of NLRP3 and CRT conducted for each treatment setting. (e) LDH release assay using culture supernatants carried out under different regimens (n = 4). ELISA quantification of (f) IL‐1β and (g) IL‐18 in supernatants measured within the designated groups (n = 4). (h) Transwell assays for migration and Matrigel‐coated invasion performed in parallel across different conditions. (i) Flow cytometry analysis and (j) related quantitative graphs of cell mortality rates in each group (n = 3). (k) Live/dead staining was employed to distinguish between viable and non‐viable cells across different treatments. (l) Immunofluorescence imaging of HMGB1 and (m) CRT acquired for all labeled groups. (n) Measurement of extracellular ATP levels in supernatants assayed under different conditions (n = 4). (o) Schematic workflow of the sequential in vitro immune‐activation assay. (p) Flow cytometry analysis of BMDCs maturation rates in different groups and (q) corresponding quantitative graphs (n = 3). (r) Flow cytometry analysis of RAW264.7 cell polarization and (s) corresponding quantification of M1‐like macrophages in each experimental group (n = 3). ELISA quantification of (t) TNF‐α and (u) IFN‐γ in supernatants measured across the indicated conditions (n = 4). Statistical analyses were performed using one‐way ANOVA followed by Tukey's multiple‐comparisons test. Significance: ns, not significant; ^*^ *p* < 0.05; ^**^ *p* < 0.01; ^***^ *p* < 0.001; ^****^ *p* < 0.0001.

Given the highly invasive nature of metastatic OS, Transwell assays were employed to assess phenotypic modulation. The results demonstrated significant suppression of 143B cell migration and invasion in the ZMG@CS group, suggesting that this treatment mitigates malignant tumor behavior by disrupting metabolic and cytoskeletal homeostasis (Figure 5h). Annexin V‐FITC/PI dual staining showed a substantially higher proportion of cell death in the ZMG@CS group (62.6%) than in the control group (5%) (Figure 5i,j), and live/dead staining also showed extensive cell death (Figure 5k). These findings support the potent anti‐tumor activity of this nanoplatform through the integration of metabolic collapse and inflammatory death signaling. To further clarify the relative contributions of different death programs, we performed functional inhibitor assays using CCK‐8 analysis in both 143B and K7M2 cells. DTT was selected as a representative reductant to evaluate disulfide‐stress‐related cytotoxicity, whereas VX‐765 was used to assess caspase‐1‐dependent pyroptosis. Fer‐1 and Nec‐1S were included in parallel to examine the possible involvement of ferroptosis and necroptosis, respectively. Compared with Fer‐1 and Nec‐1S, DTT and VX‐765 produced more evident protection against ZMG@CS‐induced loss of cell viability (Figure S8). These results indicate that ZMG@CS‐induced cell death is preferentially associated with disulfidptosis and pyroptosis rather than being primarily driven by ferroptosis or necroptosis. This functional inhibitor profile is consistent with the metabolic collapse, redox imbalance, actin cytoskeletal disruption, and inflammasome activation observed above.

To determine whether ZMG@CS enhances immunogenicity, damage‐associated molecular patterns (DAMPs) were assessed by immunofluorescence and quantitative analyses. Immunofluorescence revealed the most pronounced alteration in high mobility group box 1 protein (HMGB1) localization in the ZMG@CS group (Figure 5l). Notably, calreticulin (CRT) exposure was highest in the ZMG@CS group, as shown by WB and immunofluorescence staining (Figure 5d,m). In parallel, extracellular ATP levels increased in 143B cells following ZMG@CS treatment, further supporting induction of immunogenic cell death (ICD) (Figure 5n). To further validate the immune microenvironment remodeling effect of ZMG@CS, sequential immune activation experiments were conducted according to the workflow depicted in Figure 5o. Bone marrow‐derived dendritic cells (BMDCs) exhibited a higher maturation rate (43.8%) after exposure to ZMG@CS‐pretreated tumor cells, representing a 2.4‐fold increase compared with the control group (Figure 5p,q). Notably, RAW264.7 cells also exhibited a shift toward an M1‐like phenotype, showing a 10.3‐fold increase compared to the PBS group (Figure 5r,s). Correspondingly, TNF‐α and IFN‐γ levels in the supernatant were higher in the ZMG@CS group (Figure 5t,u). Therefore, lysosomal Zn‐driven pyroptosis not only directly promotes tumor cell elimination but also establishes a highly immunogenic milieu that recruits and promotes antigen‐presenting cells activation and M1‐like macrophages polarization.

### 2.6 Therapeutic Molecular Mechanism

To further elucidate the molecular basis of ZMG@CS therapy, transcriptomic analysis was performed on cells from the control and ZMG@CS groups. Volcano plots identified 10,413 differentially expressed genes in the ZMG@CS group compared with the control group (Figure 6a). The clustering heatmap revealed coordinated changes in multiple genes associated with signal transduction and immune regulation, suggesting that ZMG@CS‐induced disruption of cellular homeostasis extends beyond nonspecific oxidative damage (Figure 6b). Notably, both HIF1A and CD274, the gene encoding programmed death‐ligand 1 (PD‐L1), were significantly downregulated. Prior studies indicate that, after nuclear translocation, HIF‐1α forms a complex with HIF‐1β and directly binds to hypoxia‐response elements within the CD274 promoter, thereby enhancing CD274 transcription [42, 43]. Consequently, PD‐L1 is often upregulated in the hypoxic TME, accompanied by reduced effector T cell infiltration and a stronger immunosuppressive microenvironment [44, 45]. Kyoto Encyclopedia of Genes and Genomes (KEGG) enrichment analysis indicated substantial enrichment in immune regulation, immune checkpoint‐related, and cell proliferation signaling pathways after ZMG@CS treatment (Figure 6c–e). Gene Ontology (GO) enrichment analysis further highlighted alterations in lysosomal membrane‐associated pathways, cellular stress responses, cytoskeletal functions, and glucose uptake pathways (Figure 6f,g). Gene Set Enrichment Analysis (GSEA) showed significant negative enrichment of lysosomal membrane‐associated gene sets following ZMG@CS treatment, suggesting broad suppression of transcriptional programs related to lysosomal membrane structure and integrity. This finding is consistent with the experimentally observed phenotypes of lysosomal stress, zinc overload, and membrane rupture (Figure 6h). In addition, the immune checkpoint pathway in cancer also exhibited significant negative enrichment (Figure 6i), suggesting that endogenous stress signaling induced by ZMG@CS can attenuate programs associated with immune evasion.

**FIGURE 6:**
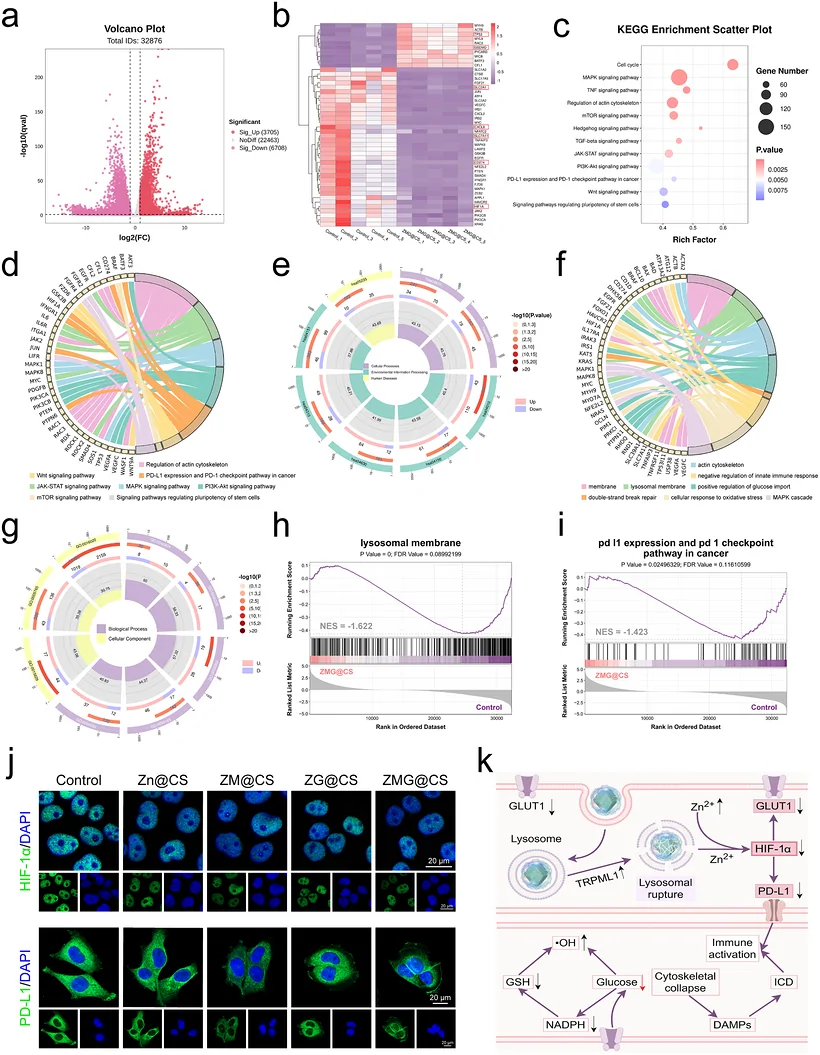
(a) Volcano plot depicting differential gene expression between the indicated conditions. (b) Hierarchical clustering heatmap showing relative expression patterns of differentially expressed genes across the compared groups (n = 5). (c) KEGG pathway enrichment scatter plot in which dot size reflects gene counts and color indicates enrichment significance. KEGG pathway enrichment analysis after different treatments is shown as (d) a chord diagram and (e) a Circos plot. GO enrichment analysis after different treatments is presented as (f) a chord diagram and (g) a Circos plot. (h,i) GSEA plots for representative gene sets. (j) Immunofluorescence imaging of HIF‐1α and PD‐L1 in cells subjected to different treatment conditions. (k) Schematic illustration summarizing the regulatory framework investigated in this study based on transcriptomic analyses and established pathway knowledge.

Mechanistic validation was further supported by immunofluorescence. Compared with the other treatments, HIF‐1α fluorescence intensity in 143B cells was significantly reduced after ZMG@CS treatment, suggesting that glucose metabolism inhibition coupled with zinc overload effectively suppresses HIF‐1α activity and stability (Figure 6j). Meanwhile, PD‐L1 fluorescence intensity decreased substantially, with weakened membrane‐ associated distribution, consistent with GSEA results. PD‐L1 downregulation also provides a mechanistic basis for combining ZMG@CS with immune checkpoint blockade in vivo. TRPML1 activation enhances lysosomal ion flux and promotes lysosomal rupture and autophagy inhibition under Zn overload, which aligns with GSEA results (Figure S9).

Because the evidence for the TRPML1/HIF‐1α/GLUT1 axis was initially derived mainly from ML‐SA5 and ZnCl_2_ co‐treatment, we next examined whether TRPML1 depletion would affect the response of 143B cells to ZMG@CS. Three siRNAs targeting TRPML1 were first examined, and siTRPML1‐3 was selected for subsequent experiments because it produced the most pronounced reduction in TRPML1 protein expression (Figure S10a and Table S3). In ZMG@CS‐treated cells, TRPML1 knockdown attenuated the decrease in HIF‐1α and GLUT1 protein levels, as determined by western blotting (Figure S10b). GLUT1 immunofluorescence showed a consistent pattern, with stronger GLUT1 staining retained in the ZMG@CS + siTRPML1 group than in cells treated with ZMG@CS alone (Figure S10c). Functionally, TRPML1 silencing increased intracellular glucose content and ATP levels in ZMG@CS‐treated cells, suggesting that loss of TRPML1 partly relieved ZMG@CS‐induced blockade of glucose metabolism and energy depletion (Figure S10d,e). TRPML1 knockdown also reduced LDH release and mitigated the pyroptosis‐like morphological changes observed by bright‐field microscopy (Figure S10f,g). In addition, phalloidin staining showed less severe F‐actin disorganization after TRPML1 knockdown (Figure S10h). These findings indicate that TRPML1 is functionally involved in ZMG@CS‐mediated HIF‐1α/GLUT1 suppression. Under zinc overload, TRPML1 activation promotes lysosomal dysfunction and reduces HIF‐1α/GLUT1 signaling, thereby impairing glucose uptake, ATP production, NADPH generation, and GSH regeneration. This metabolic disruption further contributes to pyroptosis and disulfidptosis‐associated cytoskeletal collapse (Figure 6k).

### 2.7 In Vivo Anti‐Tumor Efficacy and Mechanistic Validation

The specific accumulation of the ZMG@CS nanoplatform within tumors was evaluated in K7M2 tumor‐bearing BALB/c mice. To visualize ZMG@CS nanoparticle distribution within tumors, Cy5.5‐labeled ZMG@CS was prepared. When tumors reached approximately 100 mm^3^, Cy5.5‐ZMG@CS was intravenously administered, and its biodistribution was evaluated using an in vivo imaging system (IVIS). At 2 h post‐injection, only faint background signals were detected in the tumor region. Over time, the fluorescence intensity of Cy5.5‐ZMG@CS gradually increased in the tumor region, reaching a peak at 12 h post‐injection (Figure S11a). At 12 h after administration, mice were sacrificed, and tumors and major organs, including the heart, liver, spleen, lung, and kidney, were harvested. Compared with the liver and kidney, tumors exhibited substantially higher ZMG@CS accumulation (Figure S11b). These results suggest that, in addition to passive delivery through the enhanced permeability and retention (EPR) effect, ZMG@CS can actively target tumors by binding to CD44 receptors overexpressed on OS cells.

To systematically evaluate the in vivo anti‐tumor efficacy and biosafety of ZMG@CS, mice received multiple intravenous administrations according to a predefined schedule with longitudinal tumor monitoring (Figure 7a). Hemolysis assays demonstrated that ZMG@CS maintained an extremely low hemolysis rate (<5%) even at higher concentrations, indicating excellent blood compatibility (Figure S12). During treatment, body weights remained stable without notable abnormalities, suggesting good biocompatibility of the nanomedicine system (Figure 7b). Furthermore, H&E staining of major organs showed no significant tissue damage (Figure S13), and blood biochemical and hematological parameters remained within normal ranges (Figure S14), confirming the favorable in vivo biosafety of ZMG@CS. To evaluate the potential systemic toxicity associated with Zn delivery, zinc contents in major organs and tumor tissues were quantified by ICP‐MS at the end of treatment. Compared with the control group, ZMG@CS treatment markedly increased Zn accumulation in tumor tissues, whereas only minor changes were detected in major organs (Figure S15). These results indicate preferential Zn enrichment in tumor tissues without excessive deposition in major organs, further supporting the favorable in vivo biocompatibility of ZMG@CS. Compared with controls, ZMG@CS significantly inhibited tumor growth, as reflected by a markedly reduced tumor volume growth rate (Figure 7c,d). Endpoint tumor weights were also markedly decreased (Figure 7e; Figure S16), and Kaplan–Meier analysis showed an overall survival difference among the treatment groups (Figure 7f), supporting the enhanced in vivo anti‐tumor efficacy of ZMG@CS. Histological analysis further validated these therapeutic effects. H&E and Ki67 staining showed suppressed proliferation, whereas TUNEL staining revealed increased cell death after ZMG@CS treatment (Figure 7g). Importantly, HIF‐1α protein expression was significantly downregulated in tumor tissues, consistent with the in vitro mechanistic findings described above (Figure 7h; Figure S17). Meanwhile, histopathological examination revealed that ZMG@CS markedly upregulated cleaved caspase‐1 expression in tumor tissues while significantly downregulating GLUT1 levels (Figure 7h), suggesting the concurrent in vivo occurrence of inhibited glucose metabolism and pyroptosis.

**FIGURE 7:**
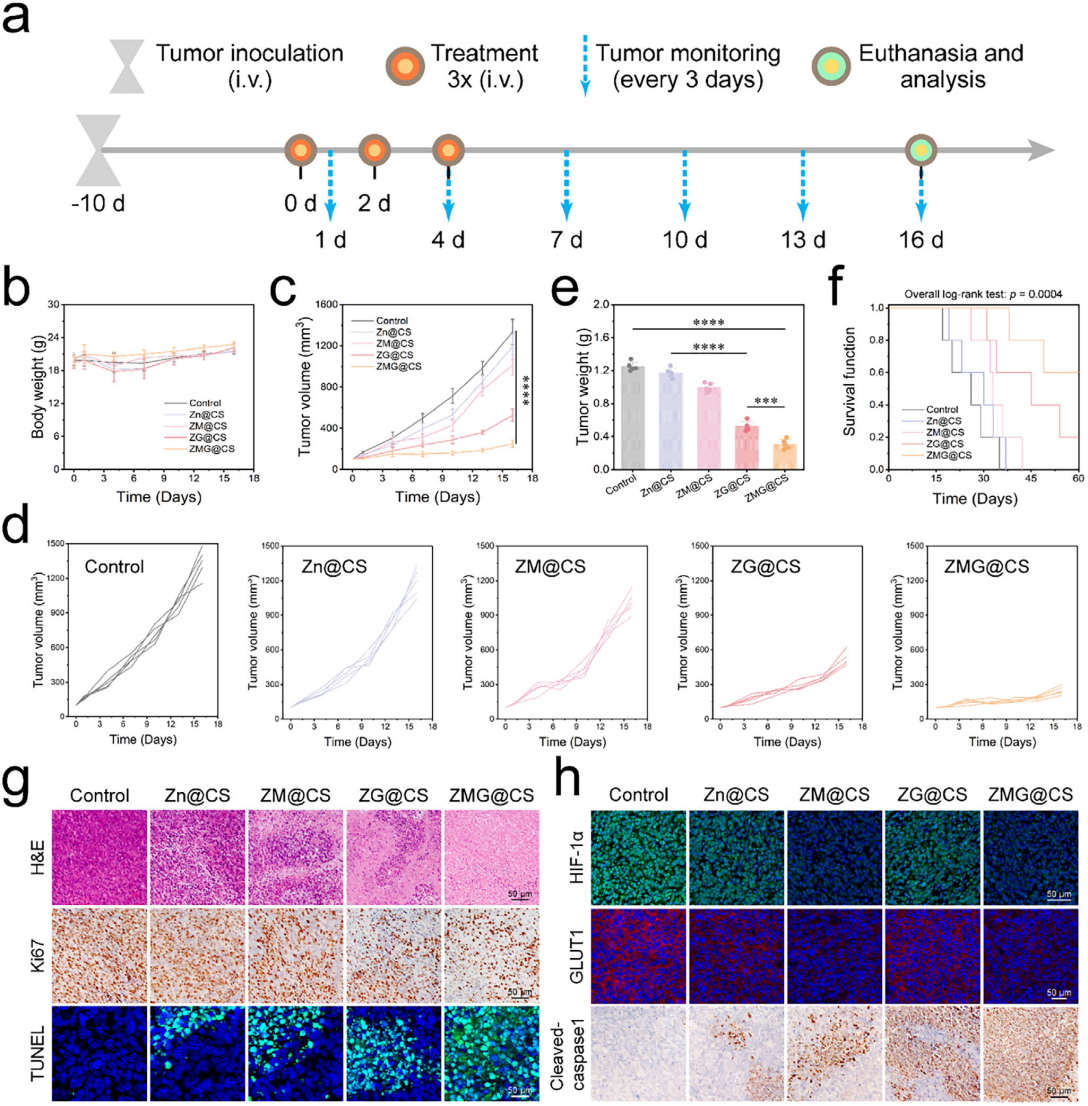
(a) Schematic illustration of the in vivo protocol. (b) Body weight monitored during the treatment period (n = 5). (c,d) Longitudinal assessment of tumor volume over time (n = 5). (e) Endpoint tumor mass measured after excision (n = 5). (f) Kaplan–Meier survival curves (n = 5). (g) Representative tumor sections evaluated by H&E staining, Ki67 immunohistochemistry, and TUNEL staining. (h) Representative tumor sections analyzed by immunofluorescence staining for HIF‐1α and GLUT1 and by immunohistochemistry for cleaved caspase‐1. Statistical analyses were performed using one‐way ANOVA followed by Tukey's multiple‐comparisons test. Tumor growth curves were analyzed by two‐way repeated‐measures ANOVA, and Kaplan–Meier survival curves were compared using the log‐rank (Mantel‐Cox) test. Significance: ns, not significant; ^*^ *p* < 0.05; ^**^ *p* < 0.01; ^***^ *p* < 0.001; ^****^ *p* < 0.0001.

### 2.8 ZMG@CS‐Mediated Immune Response

As illustrated in Figure 8a, ZMG@CS initiated an anti‐tumor immune response associated with the induction of ICD. ELISA measurements showed that serum IL‐18 and IL‐1β levels increased by 7.8‐fold and 13‐fold, respectively, in the ZMG@CS group compared with controls, consistent with intratumoral pyroptosis (Figure 8b,c). Immunofluorescence analysis of tumor sections revealed that ZMG@CS treatment significantly promoted extracellular release of HMGB1 and surface exposure of CRT (Figure 8d). These findings indicate that ZMG@CS induces robust ICD within the TME, thereby laying the groundwork for subsequent immune activation. To more directly evaluate local immune changes within tumor tissues, we further analyzed immune cell infiltration by flow cytometry and immunofluorescence staining. Flow cytometric analysis showed that ZMG@CS treatment increased the proportion of mature DCs within tumor tissues (Figure S18a,b). Meanwhile, the proportions of CD8^+^ and CD4^+^ T cells within tumor tissues increased to 12.0% and 12.7%, respectively, after ZMG@CS treatment (Figure S18c–f). Consistently, immunofluorescence staining of tumor sections showed increased infiltration of CD8^+^ and CD4^+^ T cells (Figure S18g,h). These results indicate that ZMG@CS promotes local immune activation within tumor tissues.

**FIGURE 8:**
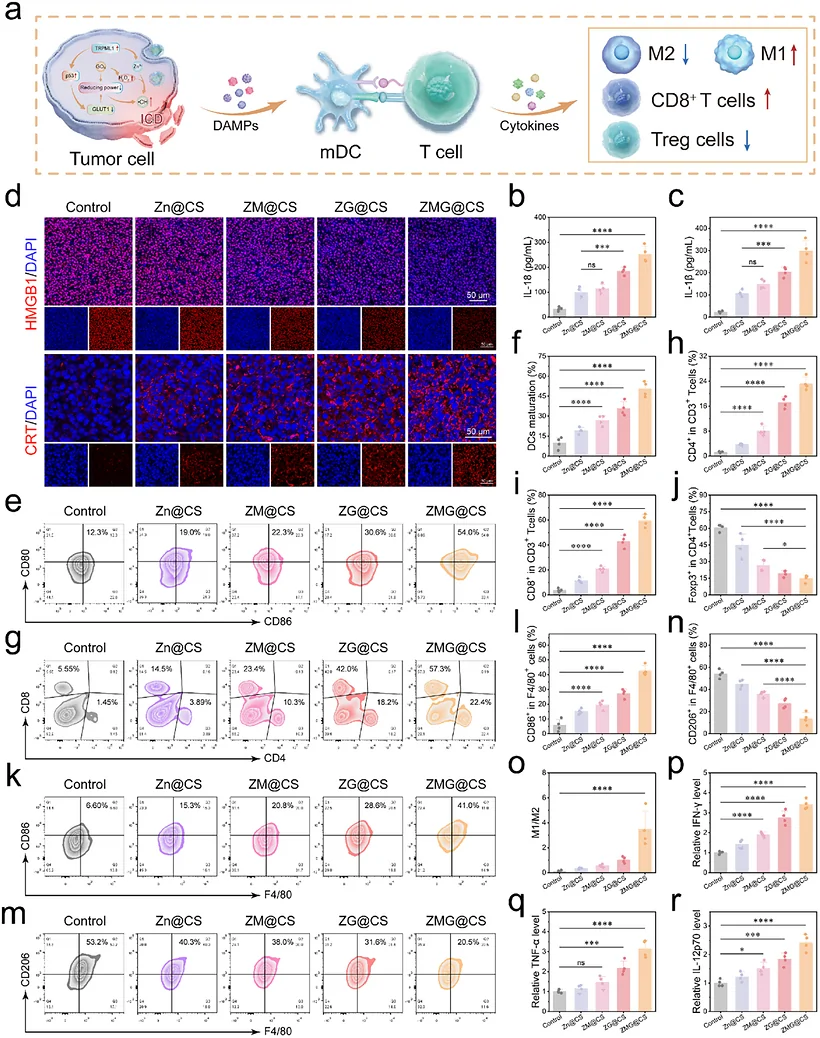
(a) Schematic illustration of the immunomodulatory mechanism of ZMG@CS. Serum inflammatory cytokines (b) IL‐18 and (c) IL‐1β were quantified using ELISA (n = 4). (d) Representative immunofluorescence images of HMGB1 and CRT in various groups. (e,f) Quantitative analysis of DCs maturation by flow cytometry (n = 4). (g–i) Flow cytometry analysis of the percentages of CD4^+^ and CD8^+^ T cells within splenic CD3^+^ T cells (n = 4). (j) Flow cytometric analysis of the proportion of Tregs among splenic CD3^+^ T cells (n = 4). k,l) Flow cytometry and quantification of M1‐like macrophages in the spleen (n = 4). (m–o) Flow cytometry quantification of M2‐like macrophages in the spleen and calculation of the M1 to M2 ratio (n) = 4). Quantitative analysis of immune‐activating cytokines (p) IFN‐γ, (q) TNF‐α, and (r) IL‐12p70 in serum using ELISA (n = 4). Statistical analyses were performed using one‐way ANOVA followed by Tukey's multiple‐comparisons test. Significance: ns, not significant; ^*^ *p* < 0.05; ^**^ *p* < 0.01; ^***^ *p* < 0.001; ****p < 0.0001.

Having observed local immune activation in tumor tissues, we further examined whether ZMG@CS treatment was accompanied by systemic immune activation in the spleen. Flow cytometry showed that the proportion of mature dendritic cells in the spleen increased to 50.4% after ZMG@CS treatment (Figure 8e,f), supporting effective innate immune activation and improved antigen presentation capacity. In addition, ZMG@CS altered splenic T cell subset composition, reflecting modulation of adaptive immunity. Relative to controls, ZMG@CS treatment significantly increased the proportions of CD4^+^ T cells (23.2%) and CD8^+^ T cells (59.5%) among CD3^+^ T cells (Figure 8g–i). Notably, the proportion of regulatory T cells (Tregs) decreased to 14.9% after ZMG@CS treatment (Figure 8j; Figure S19), indicating effective reversal of immunosuppression.

Simultaneously, ZMG@CS effectively induced polarization of tumor‐associated macrophages (TAMs) toward a proinflammatory M1 phenotype. Flow cytometric profiling revealed an increased fraction of M1‐like macrophages (42.7%) accompanied by a reduced fraction of M2‐ fraction (13.7%), resulting in an M1/M2 ratio shift from 0.11 to 3.49 (Figure 8k–o). Consistent with these immune compositional changes, proinflammatory cytokine secretion increased markedly (Figure 8p–r), including IFN‐γ (around 3.42‐fold), TNF‐α (around 3.15‐fold), and IL12p70 (approximately 2.42‐fold). In summary, ZMG@CS promotes the conversion of “cold” tumors into “hot” tumors by inducing potent ICD, simultaneously activating innate and adaptive immunity, and synergistically reshaping the tumor immune microenvironment.

### 2.9 ZMG@CS Evokes Systemic Immune Response

Pulmonary metastasis is the primary cause of mortality in OS. To determine whether ZMG@CS‐mediated systemic immune responses can achieve systemic tumor rejection, we further evaluated therapeutic efficacy in a lung metastasis model and examined the synergistic effects of ZMG@CS with immune checkpoint blockade (Figure 9a). Tumor growth analysis showed that the combination therapy produced the most pronounced tumor suppression among the treatment groups (Figure 9b). Kaplan–Meier analysis further showed a significant difference in overall survival among the four treatment groups, as determined by the log‐rank test (Figure 9c). Consistently, tumor histopathology staining revealed markedly suppressed cell proliferation and the most pronounced disruption of tumor architecture in the G4 group (Figure 9d). To evaluate immune memory activation following the strong immune response triggered by ZMG@CS, we analyzed the proportions of effector memory T cells (Tem, CD62L^−^CD44^+^) and central memory T cells (Tcm, CD62L^+^CD44^+^) in the spleen. Flow cytometry indicated that combination therapy significantly increased the proportion of memory T cells and elevated the percentage of CD8^+^ T cells among CD3^+^ T cells (46.4%), suggesting successful induction of durable and effective anti‐tumor T cell immunity (Figure 9e–g). To further assess whether the local anti‐tumor immune response was associated with a memory‐related T cell phenotype, we analyzed memory CD8^+^ T cell subsets in tumor tissues by flow cytometry. Compared with the control group, ZMG@CS increased the proportions of both Tem and Tcm cells among intratumoral CD8^+^ T cells. This increase was more evident in the ZMG@CS + αPD‐L1 group, which showed the highest levels of both subsets (Figure S20). These results support the presence of a memory‐associated T cell phenotype within tumor tissues after combination treatment.

**FIGURE 9:**
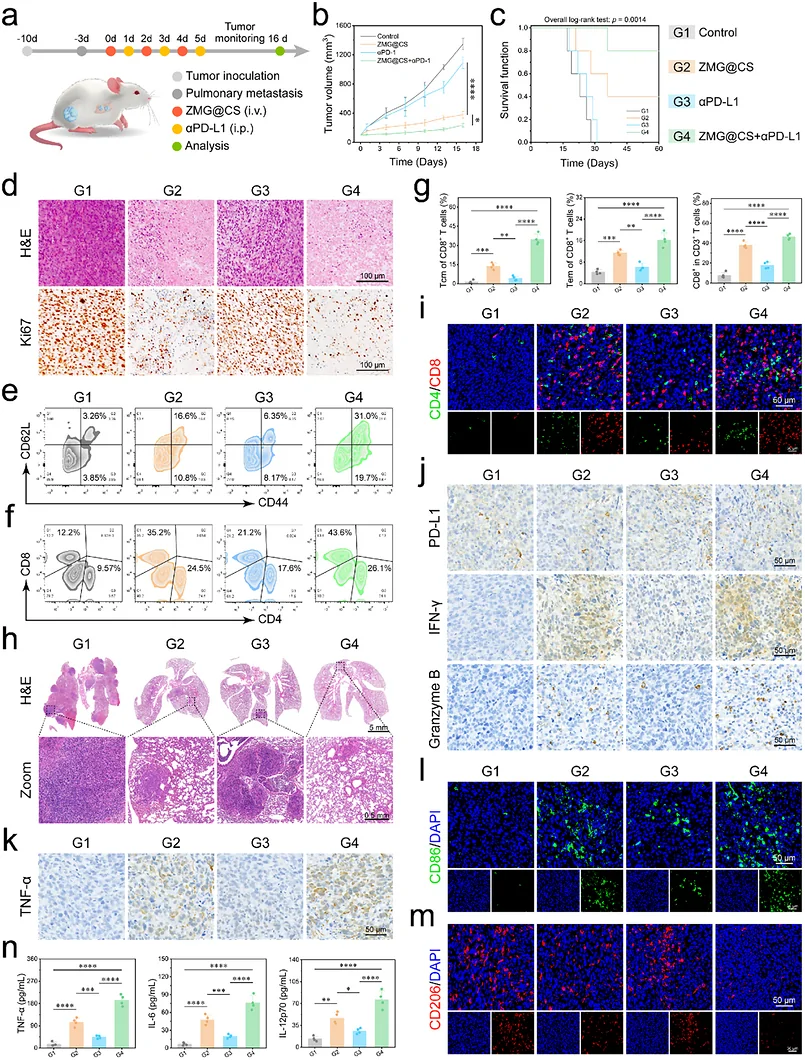
(a) Schematic of the in vivo study timeline. (b) Tumor growth monitoring over time. (c) Kaplan–Meier survival curves. (d) Representative tumor sections analyzed by H&E staining and Ki67 immunohistochemistry. (e) Flow cytometric analysis of the distribution of memory T cell subsets in CD8^+^ T cells. (f) Flow cytometry analysis of CD4^+^ and CD8^+^ T cells among splenic CD3+ T cells. (g) Quantification of central memory and effector memory T cells among CD8^+^ T cells and of CD8^+^ T cells among CD3^+^ T cells (n = 4). (h) Representative H&E staining of whole lungs with magnified views. (i) Representative immunofluorescence staining of CD4 and CD8 in different groups. (j,k) Representative immunohistochemical staining of PD‐L1, IFN‐γ, granzyme B, and TNF‐α in tumor sections. Representative immunofluorescence staining of (l) CD86 and (m) CD206 in tumor sections. (n) Quantification of serum cytokine levels (TNF‐α, IL‐6, and IL‐12p70) measured by ELISA (n = 4). Statistical analyses were performed using one‐way ANOVA followed by Tukey's multiple‐comparisons test. Tumor growth curves were analyzed by two‐way repeated‐measures ANOVA, and Kaplan–Meier survival curves were compared using the log‐rank (Mantel‐Cox) test. Significance: ns, not significant; ^*^ *p* < 0.05; ^**^ *p* < 0.01; ^***^ *p* < 0.001; ^****^ *p* < 0.0001.

In the pulmonary metastasis assessment, the G4 group exhibited the strongest suppression of metastasis. Whole lung images and magnified views revealed markedly reduced numbers and areas of metastatic nodules in the G4 group, indicating that the synergistic effect of ZMG@CS and αPD‐L1 conferred significant advantages in inhibiting distant metastasis (Figure 9h). Immunofluorescence analysis of tumor tissues showed increased infiltration of CD4^+^ and CD8^+^ T cells (Figure 9i). Immunohistochemistry showed that PD‐L1 staining did not decrease markedly, whereas IFN‐γ, granzyme B, and TNF‐α signals increased substantially, suggesting enhanced cytotoxic T‐cell activity after combination treatment (Figure 9j,k). In addition, combination therapy significantly remodeled the tumor immune microenvironment. Immunofluorescence analysis of tumors in the G4 group revealed a notable increase in CD86^+^ proinflammatory macrophages and a significant decrease in CD206^+^ anti‐inflammatory macrophages (Figure 9l,m), accompanied by substantial upregulation of key proinflammatory serum cytokines, including TNF‐α, IL‐6, and IL‐12p70 (Figure 9n). Collectively, these findings demonstrate that ZMG@CS contributes to local and systemic anti‐tumor immune activation through lysosomal Zn‐driven disulfidptosis and pyroptosis. In combination with immune checkpoint blockade, ZMG@CS amplified T cell‐mediated tumor killing and improved the control of pulmonary metastasis.

## 3 Conclusions

In summary, we developed a TRPML1‐activation‐based cascade nanoplatform (ZMG@CS) that integrates endogenous and exogenous zinc signaling to disrupt glucose metabolism reprogramming in highly metastatic OS cells, ultimately reshaping the tumor immune microenvironment. Within the acidic lysosomal environment, ZMG@CS couples ion homeostasis disruption with glucose metabolism suppression via the TRPML1/HIF‐1α/GLUT1 axis. GOx provides acidic gating and substrate supply, while Zn single‐atom sites further amplify POD‐like catalysis to promote ROS generation, driving rapid exhaustion of the NADPH/GSH axis and collapse of the actin cytoskeleton, thereby triggering disulfidptosis. Concurrently, TRPML1 activation promotes cytoplasmic Zn^2+^ overload, induces lysosomal rupture, and activates NLRP3/caspase‐1‐dependent immunogenic pyroptosis. Subsequently, pyroptotic tumor cells release abundant inflammatory mediators, thereby driving ICD and reversing the immunosuppressive microenvironment. Across multiple in vitro and in vivo models, ZMG@CS significantly suppressed tumor growth and lung metastasis. Combination with anti‐PD‐L1 further strengthened long‐lasting T cell‐mediated anti‐tumor immune memory. Although ZMG@CS showed therapeutic effects in both human‐ and mouse‐derived osteosarcoma cell lines, these established models do not fully recapitulate the heterogeneity and clinical complexity of patient‐derived tumors. Future studies using patient‐derived OS cells, organoids, or more clinically relevant tumor models are warranted to further validate the translational potential and broader applicability of ZMG@CS. Collectively, this study proposes a therapeutic strategy that integrates metabolic suppression and ion homeostasis disruption through programmable lysosomal Zn overload, providing a potential approach for OS immunotherapy.

## 4 Experimental Section

### 4.1 Preparation of ZMG@CS

Zn(NO_3_)_2_·6H_2_O (2.38 g) and zinc acetylacetonate (100 mg) were dissolved in 100 mL methanol and homogenized by ultrasonication. In parallel, 2‐methylimidazole (2.77 g) was dissolved in another 100 mL methanol. The two solutions were mixed at room temperature and stirred for 2 h. The product was then isolated via centrifugation, washed thrice with methanol, and dried at 60°C for 24 h to yield ZIF‐8.

Subsequently, ZIF‐8 (5 g) was subjected to pyrolysis at 800°C for 2 h under an argon atmosphere with a heating rate of 10°C min^−1^, resulting in the formation of hZn‐NC. GOx (10 mg) and ML‐SA5 (20 mg) were co‐dissolved in 0.5 mL DMSO, and deionized water was added to a final volume of 20 mL to prepare a mixed solution, which was homogenized by ultrasonication. Then, hZn‐NC (50 mg) was added to the above solution, sonicated for 10 min, and stirred at room temperature for 24 h. The mixture was centrifuged and washed with deionized water three times, followed by vacuum freeze‐drying to afford hZn‐NC@ML‐SA5@GOx (denoted as ZMG).

To fabricate ZMG@CS nanoparticles, ZMG (15 mg) and CS (10 mg) were dispersed in deionized water and stirred at room temperature for 24 h. The product was collected by centrifugation and washed with deionized water to obtain the final product.

### 4.2 pH Responsiveness of ZMG@CS

To evaluate the pH‐responsive behavior of ZMG@CS, the release of Zn^2+^ from the nanomaterial was examined under simulated environments with different pH values. Briefly, ZMG@CS (1 mg) was dispersed in 2 mL of PBS buffer adjusted to pH 5.5, 6.5, or 7.4, and incubated at 37°C with shaking. At predetermined time points (2, 4, 6, 12, and 24 h), supernatants were collected, and the samples were analyzed for Zn^2+^ concentration using inductively coupled plasma mass spectrometry (ICP‐MS).

### 4.3 Evaluation of Catalytic Activity of ZMG@CS

To evaluate the in vitro •OH production, various concentrations of ZMG@CS (0, 10, 15, 25, 50, and 100 µg mL^−^^1^) were incubated with methylene blue (MB, 15 µg mL^−^^1^) and H_2_O_2_ (15 mm) at 37°C for 30 min. The degradation of MB was evaluated by monitoring the decrease in its characteristic absorbance at 665 nm. In addition, the •OH‐generating capacity of ZMG@CS was evaluated across different pH conditions (pH 4.5, 5.5, 6.5, and 7.4). Furthermore, the steady‐state kinetics of ZMG@CS were assessed using the colorimetric reaction between 3,3′,5,5′‐tetramethylbenzidine (TMB) and H_2_O_2_ as a model system. Briefly, TMB (1 mm) was mixed with ZMG@CS and H_2_O_2_ solutions at various concentrations (0.1, 0.5, 1, 2.5, 5, and 10 mm), and the absorbance at 652 nm was measured using a UV–vis spectrophotometer to monitor the formation of oxidized TMB. Apparent kinetic parameters, including the maximum reaction rate (Vmax) and the Michaelis constant (Km), were calculated by fitting the data to the Michaelis–Menten equation. Finally, ESR spectroscopy was performed to provide direct evidence of radical formation. ZMG@CS was added to a system containing the spin‐trapping agent DMPO and glucose, and the characteristic radical signals were recorded to confirm that the nanocatalyst generated •OH in the presence of glucose.

### 4.4 Cell Culture

Human osteosarcoma 143B cells, murine osteosarcoma K7M2 cells, and RAW264.7 cells were cultured in high‐glucose Dulbecco's modified Eagle's medium (DMEM) supplemented with 10% fetal bovine serum (FBS) and 1% penicillin/streptomycin, and maintained at 37°C in a humidified incubator with 5% CO_2_. Normal human osteoblasts hFOB 1.19 cells were cultured in the same medium and maintained at 34°C in a humidified incubator with 5% CO_2_.

### 4.5 Cellular Energy‐Related Evaluation

Cellular energy metabolism was evaluated by measuring intracellular glucose content and ATP levels to determine the effects of each treatment. 143B and K7M2 cells were seeded overnight in 6‐well plates and then treated for 12 h with PBS, Zn@CS, ZM@CS, ZG@CS, or ZMG@CS. Post‐treatment, the cells were washed twice with PBS, followed by the addition of cell lysis buffer to each well to extract intracellular contents. The supernatants obtained from centrifuged lysates were used for glucose and ATP measurements. Intracellular glucose was measured via the O‐toluidine method, and ATP levels were assessed using a luminescence‐based assay kit.

### 4.6 Intracellular Redox Homeostasis Assessment

First, intracellular ROS levels were quantified using the fluorescent indicator DCFH‐DA. Tumor cells were plated in culture dishes and exposed to the indicated nanomaterials (as defined in the grouping scheme) for 12 h. Cells were subsequently incubated with 1 mL DCFH‐DA working solution and incubated at 37°C for 30 min, followed by two rinses with PBS. Fluorescence signals were acquired immediately using confocal laser scanning microscopy (CLSM).

Since intracellular reducing power is closely related to NADPH and cysteine, the levels of NADPH and cysteine were further measured in each treatment group. After treatment, 143B and K7M2 cells were collected, and NADPH levels were quantified using a commercial NADP^+^/NADPH assay kit. Briefly, NADPH and NADP^+^ were extracted and separated using the extraction buffer, followed by an enzyme cycling reaction in which NADPH was oxidized to generate a colorimetric signal. The absorbance was recorded at 450 nm. In parallel, intracellular cysteine was determined using a cysteine assay kit based on a reduction‐colorimetric method. Absorbance at 600 nm was measured in the cell lysates.

To further characterize the intracellular redox state, GSH and GSSG were measured, and the GSSG/GSH ratio was calculated. Treated 143B and K7M2 cells were collected and mixed with the protein removal reagent M. After centrifugation, one portion of the supernatant was used directly to determine total GSH, whereas another portion was first incubated with a GSH scavenger solution to mask GSH and then used for GSSG determination. GSH content was calculated as total GSH minus twice the GSSG content, and the GSSG/GSH ratio was then obtained.

### 4.7 Pyroptosis Pathway Evaluation In Vitro

To elucidate the pyroptotic pathway induced by ZMG@CS, inflammasome activation and downstream execution events were examined at the cellular level. Tumor cells from each treatment group were collected, and total proteins were extracted for western blot analysis of NLRP3 expression and the cleavage of caspase‐1 and GSDMD. Protein concentrations in the clarified lysates were determined using a BCA assay. Equal amounts of protein were separated by SDS‐PAGE and transferred onto PVDF membranes. The membranes were incubated with anti‐NLRP3, anti‐caspase‐1, and anti‐GSDMD antibodies to detect corresponding protein expression, followed by visualization using an enhanced chemiluminescence (ECL) system. In parallel, LDH release was quantified using a commercial assay kit, and the secretion of IL‐18 and IL‐1β was measured by ELISA.

### 4.8 Immunofluorescence

To assess the release of DAMPs and the localization changes of key pathway proteins induced by ZMG@CS treatment, immunofluorescence staining was performed. After treatment for 12 h, 143B cells were fixed with 4% paraformaldehyde at room temperature for 10 min. The cells were then permeabilized with 0.1% Triton X‐100 for 10 min and washed three times with PBS. Following permeabilization, the cells were incubated with immunofluorescence blocking solution for 30 min. After blocking, the cells were incubated with primary antibody working solution at 4°C overnight. The next day, the cells were washed with PBS and incubated with fluorescently labeled secondary antibody at room temperature for 1 h in the dark. After counterstaining the nuclei with DAPI for 5 min, fluorescence signals were observed using CLSM.

### 4.9 Anti‐tumor Efficacy Evaluation

To evaluate the overall cytotoxic effect of ZMG@CS, live/dead staining and Annexin V‐FITC/PI apoptosis assays were performed. For live/dead staining, viable and dead cells were distinguished using calcein‐AM/PI dual staining. After 12 h treatment, 143B cells were washed and incubated with working solution containing Calcein‐AM and PI at 37°C for 30 min in the dark. The cells were then observed and imaged using an inverted fluorescence microscope. In addition, the proportion of dead cells was quantified by flow cytometry. Briefly, 143B cells from each treatment group were harvested by trypsinization, collected by centrifugation, and resuspended in binding buffer. Annexin V‐FITC and PI were added, and the samples were incubated for 15 min at room temperature in the dark, followed by analysis on a flow cytometer.

### 4.10 Transcriptomic Analysis

Control and ZMG@CS‐treated 143B cells were harvested by trypsinization, washed, and centrifuged. TRIzol reagent was then slowly added to the cell pellets at a density of 5 × 10^6^ cells/mL, followed by gentle pipetting to ensure complete mixing. The lysates were carefully transferred into nuclease‐free cryovials and immediately immersed in liquid nitrogen for 30 min. The frozen samples were then packed in dry ice and transported to LC‐Bio Technologies (Hangzhou) Co., Ltd. for transcriptomic sequencing. Five biological replicates were included in each group. After data acquisition, Gene Ontology (GO) and Kyoto Encyclopedia of Genes and Genomes (KEGG) enrichment analyses were performed to investigate the biological functions and pathways associated with the differentially expressed genes. Differential expression and enrichment analyses were performed using the standard RNA‐seq analysis pipeline provided by LC‐Bio Technologies (Hangzhou) Co., Ltd.

### 4.11 Tumor Model

All animal experiments were approved by the Medical Ethics Committee of Sichuan Provincial People's Hospital (2024‐661). K7M2 cells were cultured in vitro in advance and confirmed to be in good condition. To establish a subcutaneous osteosarcoma model, female BALB/c mice (5–6 weeks old) were inoculated subcutaneously in the right hind limb with 100 µL of K7M2 cell suspension (1 × 10^6^ cells/mouse). When the tumor volume reached approximately 100 mm^3^, tumor‐bearing mice were randomly assigned to five treatment groups: Control, Zn@CS, ZM@CS, ZG@CS, and ZMG@CS, with 5 mice in each group unless otherwise stated. Treatments were administered according to the schedule described in the corresponding figure legends. Tumor length (L) and width (W) were measured every 3 days using a digital caliper, and tumor volume was calculated as *V* = *(L × W^2^)/2*. Body weight was monitored throughout the treatment period. At the experimental endpoint, mice were euthanized, and tumors and major organs were collected for subsequent analyses. Humane endpoint criteria included excessive tumor burden, ulceration, severe body weight loss, impaired mobility, or other signs of distress.

### 4.12 In Vivo Anti‐Tumor Effect

When tumors reached approximately 100 mm^3^, mice were subjected to fluorescence imaging and treatment. Treatments were administered every 2 days for a total of three doses. Tumor size and body weight were recorded every 3 days throughout the treatment period. The experiment was terminated on day 16, and mice were euthanized under anesthesia. Tumors were excised, weighed, and processed for histopathological staining. In addition, spleens were collected for lymphocyte isolation and immunological analysis, and serum cytokine levels were quantified by ELISA.

### 4.13 Therapeutic Effect of ZMG@CS Combined with Immune Checkpoint Blockade

To establish a K7M2 lung metastasis model, mice were intravenously injected via the tail vein with a K7M2 cell suspension (1 × 10^6^ cells per mouse) 7 days after subcutaneous inoculation. Three days later, the mice were randomly assigned to four groups: PBS, ZMG@CS, αPD‐L1, and ZMG@CS + αPD‐L1. The indicated intravenous treatments were administered by tail vein injection on days 0, 2, and 4, and the αPD‐L1 antibody (100 µg/mouse) was administered intraperitoneally on days 1, 3, and 5. Tumor volume and body weight were monitored every 3 days. On day 16, spleens and tumors were collected for analysis of memory T cells, and intratumoral immune responses were evaluated by histopathological staining of tumor tissues. In addition, serum cytokine levels were measured using ELISA kits according to the manufacturer's instructions.

### 4.14 Statistical Analysis

All statistical analyses were performed using GraphPad Prism 8. Where applicable, data were normalized to the corresponding control group before statistical analysis and graphical presentation. Data are presented as mean ± standard deviation (SD). The exact sample size (n) for each statistical analysis is specified in the corresponding figure legend. Comparisons between two groups were performed using Student's t‐test. For comparisons among multiple groups, one‐way analysis of variance (ANOVA) followed by Tukey's post hoc test was applied. Longitudinal tumor growth curves were analyzed using two‐way ANOVA, with treatment and time as the two factors. Kaplan–Meier survival curves were compared using the log‐rank (Mantel‐Cox) test. The significance level was set at α = 0.05, and *p* < 0.05 was considered statistically significant. Statistical significance is denoted as follows: ^*^ *p* < 0.05, ^**^ *p* < 0.01, ^***^ *p* < 0.001, and ^****^ *p* < 0.0001.

## Author Contributions

**Zhenxin Wang** conceived and designed the study. **Zhenxin Wang**, **Yuhang Tang**, and **Ronghao Yue** performed all the experiments. **Yong Tao** and **Jingtao Xu** analyzed the results and drafted the manuscript. **Jie Chen** and **Jinfang Xue** provided suggestions on data presentation. All authors discussed the results and provided feedback on the manuscript.

## Conflicts of Interest

The authors declare no conflicts of interest.

## Supporting information




**Supporting File**: advs76780‐sup‐0001‐SuppMat.docx.

## Data Availability

The data that support the findings of this study are available from the corresponding author upon reasonable request.
