# Supplementary material for: A High‐Loading Zn Single‐Atom Nanozyme Targets the Zn/HIF‐1α/GLUT1 Axis to Disrupt Glucose Metabolic Reprogramming and Remodel the Tumor Immune Microenvironment
Source: Adv Sci (Weinh). 2026 Jul 23;13(58):e76780. doi: 10.1002/advs.76780 (PMC13393267; doi:10.1002/advs.76780)
Supplement: Supplementary file 1 [file ADVS-13-e76780-s001.docx]

**A High-Loading Zn Single-Atom Nanozyme Targets the Zn/HIF-1α/GLUT1 Axis to Disrupt Glucose Metabolic Reprogramming and Remodel the Tumor Immune Microenvironment**

Zhenxin Wang^1^, Yuhang Tang^1^, Ronghao Yue^1^, Jingtao Xu, Yong Tao, Jie Chen, Shipeng Li, Guosheng Zhao, Jinfang Xue, Mingwei Sun*, Fenglei Gao*, Yunsheng Ou*, Yang Wang*

Zhenxin Wang, Yuhang Tang, Ronghao Yue, Jingtao Xu, Yong Tao, Yunsheng Ou

Department of Orthopaedic Surgery,

The First Affiliated Hospital of Chongqing Medical University,

Chongqing Medical University,

Chongqing 400016, China.

Email: ouyunsheng2001@163.com (Y. Ou).

Shipeng Li, Jinfang Xue, Mingwei Sun, Yang Wang

Department of Emergency Medicine Center,

Sichuan Provincial People's Hospital,

University of Electronic Science and Technology of China,

Chengdu, Sichuan 610065, China.

Email: 653176424@qq.com (M. Sun), young0416@163.com (Y. Wang)

Fenglei Gao

Key Laboratory of New Drug Research and Clinical Pharmacy,

Xuzhou Medical University,

Jiangsu 221004, China.

Email: flgao@xzhmu.edu.cn (F. Gao)

Guosheng Zhao

Department of Orthopaedic Surgery,

The Second Affiliated Hospital of Chongqing Medical University,

Chongqing Medical University,

Chongqing 400016, China.

Jie Chen

Department of Core Laboratory,

Sichuan Provincial People's Hospital,

University of Electronic Science and Technology of China,

Chengdu, Sichuan 610065, China.

**Experimental Section**

*Materials*: The following chemicals were sourced from Aladdin Biochemical Technology Co., Ltd. (Shanghai, China): 2-Methylimidazole, zinc nitrate hexahydrate (Zn(NO_3_)_2_·6H_2_O), glucose oxidase (GOx), chondroitin sulfate (CS). ML-SA5, DTT, VX-765, Fer-1, and Nec-1S were purchased from MedChemExpress (NJ, USA). The NADP^+^/NADPH detection kit and cysteine content detection kit were provided by Elabscience Biotechnology Co., Ltd. (Wuhan, China). Live/Dead cell staining kit, DCFH-DA probe, phalloidin, mitochondrial membrane potential assay kit (JC-1 staining), Annexin V-APC/PI apoptosis detection kit, 4',6-diamidino-2-phenylindole (DAPI), high glucose Dulbecco’s modified Eagle’s medium (DMEM), and phosphate buffered saline (PBS) were purchased from KeyGen Biotech. Co., Ltd. (Nanjing, China). CCK-8 assay, lactate dehydrogenase (LDH) release assay kit, ATP assay kit, glutathione (GSH/GSSG) assay kit, and glucose assay kit were obtained from Beyotime Biotech. Inc. (Shanghai, China). Lyso-Tracker Red, acridine orange (AO), and BCECF-AM for cell imaging were also purchased from Beyotime Biotech. Inc. (Shanghai, China). All ELISA kits were purchased from Servicebio, Inc. (Wuhan, China). Primary antibodies for western blotting and immunofluorescence, including anti-NLRP3, anti-Caspase-1 and its p20 fragment, anti-Gasdermin D and its N-terminal fragment, anti-HMGB1, anti-CRT, anti-GLUT1, anti-HIF-1α, anti-PD-L1, and anti-β-Actin, were obtained from Proteintech Group, Inc. (Wuhan, China). Transwell systems were obtained from Corning Incorporated (NY, USA). Fluorescent antibodies used in flow cytometry were provided by Thermo Fisher Scientific (WLM, USA).

*Characterization*: The hydrodynamic diameter of the nanoparticles was measured by DLS to evaluate their size distribution and colloidal stability. Zeta potential analysis was performed to examine changes in surface charge before and after CS modification, thereby confirming successful coating. TEM and HAADF-STEM were used to visualize particle morphology and microstructure, and to verify the presence of atomically dispersed Zn. EDS elemental mapping was conducted to verify the uniform distribution of Zn, C, and N within the material. In addition, XRD was used to analyze the crystalline structure. After pyrolysis, an amorphous carbon-dominated structure was expected, with no diffraction peaks corresponding to metallic Zn. XPS and XAFS were further employed to determine the oxidation state and coordination environment of Zn, confirming that Zn existed as Single-Atoms coordinated by Zn–N bonds.

*GOx enzymatic activity assay*: ZMG@CS (50 μg mL^-1^) was incubated with glucose (10 mM) at 37 °C for different durations (1, 2, 4, and 6 h), and the pH of the solution was recorded using a pH meter. At each time point, the mixture was centrifuged and the supernatant was collected, followed by the addition of ABTS (5 mM) and horseradish peroxidase (HRP, 1 mg mL^-1^). After further incubation for 30 min, the absorbance at 420 nm was measured using a UV-vis spectrophotometer to confirm H_2_O_2_ production. In addition, H_2_O_2_ generation was evaluated under different pH conditions (pH 4.5, 5.5, 6.5, and 7.4).

*Cellular uptake analysis*: Cellular uptake and subcellular localization of ZMG@CS in tumor cells were quantitatively analyzed using confocal laser scanning microscopy (CLSM) and flow cytometry. ZMG@CS nanoparticles were labeled with fluorescein isothiocyanate (FITC) and then added to the culture medium of 143B and K7M2 cells at 50 μg mL^-1^ for incubation for different times (1, 2, and 4 h). After incubation, the cells were gently washed with PBS and incubated with Lyso-Tracker Red working solution (100 nM) at 37 °C for 1 h. The nuclei were then counterstained with DAPI, and fluorescence images were acquired by CLSM. For quantitative analysis, the treated cells were collected after trypsinization, resuspended in PBS, and the intracellular FITC fluorescence intensity was measured using a flow cytometer.

*In Vitro Cytotoxicity of Different Nanoparticles*: The *in vitro* cytotoxicity of the nanomaterials toward tumor cells was determined by CCK-8 assay. Briefly, 143B and K7M2 cells were plated in 96-well plates (1 × 10^4^ cells per well) and allowed to attach overnight. Cells were subsequently exposed to Zn@CS at graded concentrations (5, 10, 15, 25, 50, 100, 200, and 400 μg mL^−1^) for 24 h. CCK-8 reagent (100 μL per well) was then applied and incubated at 37 °C for 1 h, after which absorbance at 450 nm was measured using a microplate reader to calculate cell viability. In addition, we evaluated the cytotoxic effects of different concentrations of ZMG@CS (5, 10, 15, 25, 50, 100, and 150 μg mL^−1^) and different drug combinations (PBS, Zn@CS, ZM@CS, ZG@CS, ZMG@CS) on cells. To optimize the dosing ratio for combination therapy, the synergistic effect of GOx and ML-SA5 was further evaluated by varying the molar ratio of GOx to ML-SA5 (1:1, 1:2, 1:3, and 1:4) at a fixed total dose. The mixtures were added to 143B cells and incubated for 24 h, and cell viability was determined using the CCK-8 assay.

*Acridine Orange (AO) Staining*: AO staining was used to evaluate the effects of different treatments on lysosomal membrane permeability. Tumor cells were seeded in confocal dishes and cultured overnight prior to 12 h exposure to the designated formulations. Cells were then stained with 1 mL AO at 37 °C for 10 min in the dark, washed twice with PBS, and visualized by CLSM to evaluate fluorescence alterations.

*Cytoskeletal Evaluation*: To observe the effects of various treatments on cytoskeletal structure, filamentous actin (F-actin) was stained with fluorescently labeled phalloidin. OS cells were seeded in culture dishes and exposed to various drugs for 12 h. After removing the medium, cells were gently washed with pre-chilled PBS and fixed with 4% paraformaldehyde at room temperature for 15 min. The cells were permeabilized with 0.2% Triton X-100 for 5 min following three PBS washes. The cells were washed with PBS and incubated in 200 μL of phalloidin solution for 20 min. The nuclei were counterstained with DAPI for 5 min. Finally, cytoskeletal changes were imaged using CLSM.

*Measurement of Mitochondrial Membrane Potential (MMP)*: MMP was assessed using the JC-1 dye, which exhibits red fluorescence as mitochondrial aggregates under high MMP and shifts to green monomers upon membrane depolarization. 143B cells were plated in glass-bottom dishes overnight and then exposed to the indicated formulations for 12 h. The culture medium was subsequently replaced with 1 mL fresh medium supplemented with JC-1 working solution, and cells were incubated at 37 °C for 20 min. After two gentle rinses with JC-1 staining buffer, fluorescence images were acquired using CLSM.

*Evaluation of Anti-Tumor Immune Response Activation In Vitro*: A Transwell-based co-culture model was established to evaluate immune cell activation induced by tumor cells after different treatments. Briefly, pretreated tumor cells were seeded in the upper chamber, while BMDCs or undifferentiated RAW264.7 macrophages were seeded in the lower chamber. The two chambers were co-cultured for 24 h. BMDCs or RAW264.7 cells were then collected and stained with fluorophore-conjugated primary antibodies for 30 min, followed by flow cytometric analysis of DCs maturation markers (CD80 and CD86) and M1-like tumor-associated macrophage markers (F4/80 and CD86). In parallel, key cytokines (TNF-α and IFN-γ) in the co-culture supernatants were quantified by ELISA.

*In Vivo Fluorescence Imaging*: After successful establishment of the tumor-bearing mouse model, Cy5.5-labeled ZMG@CS nanoparticles were administered *via* tail vein injection. At predetermined time points after injection (2, 6, 12, and 24 h), fluorescence images were acquired using an *in vivo* imaging system (IVIS). For further verification, mice were euthanized at 12 h post-injection, and major organs (heart, liver, spleen, lung, and kidney) as well as tumors were harvested for *ex vivo* fluorescence imaging.

**Figure S1.** Standard curve of ML-SA5.

**Figure S2.** Standard curve of GOx.

**Figure S3.** EXAFS fitting of Zn foil in a) *k* space and b) R space, and EXAFS fitting of ZnO in c) *k* space and d) R space.

**Figure S4.** a) Flow cytometric quantification of ZMG@CS-FITC uptake in 143B cells (n = 3). b) Confocal fluorescence images of L929 cells incubated with ZMG@CS-FITC. c,d) Confocal imaging and flow cytometric quantification of ZMG-FITC uptake in 143B cells (n = 3). e-g) Confocal imaging and flow cytometric quantification of ZMG-FITC and ZMG@CS-FITC uptake in K7M2 cells (n = 3). Data are presented as mean ± SD.

**Figure S5.** Cell viability of different cell lines treated with graded concentrations of ZMG (n = 6). Statistical analyses were performed using one-way ANOVA followed by Tukey’s multiple-comparisons test. Significance: ns, not significant; *P < 0.05; **P < 0.01; ***P < 0.001; ****P < 0.0001.

**Figure S6.** Intracellular GSSG and GSH levels in K7M2 cells after the indicated treatments (n = 4).

**Figure S7.** Non-reducing and reducing western blot analysis of actin under different treatment conditions.

**Figure S8.** Cell viability of 143B and K7M2 cells treated with different concentrations of ZMG@CS in the presence of a) Nec-1S, b) Fer-1, c) VX-765, or d) DTT (n = 5). Statistical analyses were performed using one-way ANOVA followed by Tukey’s multiple-comparisons test. Significance: ns, not significant; *P < 0.05; **P < 0.01; ***P < 0.001; ****P < 0.0001.

**Figure S9.** GSEA enrichment plots of the a) lysosome and b) autophagy pathways comparing ZMG@CS-treated cells with control cells.

**Figure S10.** a) Western blot analysis of TRPML1 expression in 143B cells transfected with three independent TRPML1-targeting siRNAs. b) Representative western blots showing TRPML1, HIF-1α, and GLUT1 expression in 143B cells after different treatments. c) Immunofluorescence staining of GLUT1 in 143B cells. d,e) Quantification of intracellular glucose and ATP levels in 143B cells after the indicated treatments (n = 4). f) LDH release assay showing reduced ZMG@CS-induced cytotoxicity after TRPML1 knockdown (n = 4). g) Bright-field images showing cellular morphological changes under different conditions. h) Phalloidin staining of F-actin in 143B cells after the indicated treatments. Statistical analyses were performed using one-way ANOVA followed by Tukey’s multiple-comparisons test. Significance: ns, not significant; *P < 0.05; **P < 0.01; ***P < 0.001; ****P < 0.0001.

**Figure S11.** a) Time-resolved *in vivo* fluorescence biodistribution of Cy5.5-ZMG@CS in tumor-bearing mice after tail vein injection. b) *Ex vivo* fluorescence images of the major organs and tumors collected 12 h post-injection.

**Figure S12.** Hemolysis assay of ZMG@CS at different concentrations (n = 3).

**Figure S13.** Representative H&E-stained sections of major organs harvested from mice at the end of treatment.

**Figure S14.** Hematological and serum biochemical parameters in mice after completion of treatment (n = 3).

**Figure S15.** Biodistribution of Zn in major organs and tumor tissues at the end of treatment, quantified by ICP-MS (n = 5).

**Figure S16.** Representative photographs of tumor-bearing mice and excised tumors.

**Figure S17.** Western blot analysis of HIF-1α expression in tumor tissues from different groups.

**Figure S18.** a) Representative flow cytometry plots and b) quantitative analysis of mature DCs in tumor tissues after different treatments (n = 4). c) Representative flow cytometry plots and d) quantitative analysis of CD8^+^ T cells in tumor tissues (n = 4). e) Representative flow cytometry plots and f) quantitative analysis of CD4^+^ T cells in tumor tissues (n = 4). Immunofluorescence staining of g) CD4 and h) CD8 in tumor sections after different treatments. Statistical analyses were performed using one-way ANOVA followed by Tukey’s multiple-comparisons test. Significance: ns, not significant; **P* < 0.05; ***P* < 0.01; ****P* < 0.001; *****P* < 0.0001.

**Figure S19.** Flow cytometric analysis of splenic Treg cells in mice subjected to different treatments.

**Figure S20.** a) Representative flow cytometry plots showing CD44 and CD62L expression in CD8^+^ T cells from tumor tissues after different treatments. G1, Control; G2, ZMG@CS; G3, αPD-L1; G4, ZMG@CS + αPD-L1. b) Quantification of effector memory CD8^+^ T cells (Tem, CD44^+^CD62L^-^) and central memory CD8^+^ T cells (Tcm, CD44⁺CD62L⁺) in tumor tissues (n = 4). Statistical analyses were performed using one-way ANOVA followed by Tukey’s multiple-comparisons test. Significance: ns, not significant; **P* < 0.05; ***P* < 0.01; ****P* < 0.001; *****P* < 0.0001.

**Table S1.** Zn content in hZn-NC measured by ICP-OES.

| Sample | Zn content  (mg/kg) | Zn content  (%) |
| --- | --- | --- |
| hZn-NC-1 | 59205.9064 | 5.9206% |
| hZn-NC-2 | 59257.5665 | 5.9258% |
| hZn-NC-3 | 59437.5301 | 5.9438% |

**Table S2.** EXAFS fitting parameters at the Zn K-edge for various samples. (*Ѕ*_0_^2^ = 0.95 from Zn-foil)

| Sample | Shell | CN*^a^* | R*^b^* (Å) | σ^2^*^c^* (Å^2^) | ΔE_0_*^d^* (eV) | R factor |
| --- | --- | --- | --- | --- | --- | --- |
| Zn Foil | Zn-Zn | 6 | 2.64±0.01 | 0.0101 | 1.3 ± 0.6 | 0.006 |
| ZnO | Zn-O | 4 | 1.94±0.06 | 0.0043 | 2.3 ± 0.7 | 0.010 |
|  | Zn-Zn | 12 | 3.23±0.04 | 0.0065 |  |  |
| Zn-NC | Zn-N | 4.08±0.4 | 2.04±0.07 | 0.0095 | 1.2 ± 0.2 | 0.016 |

*^a^CN*: coordination numbers; *^b^R*: bond distance; *^c^σ*^2^: Debye-Waller factors; *^d^*Δ*E*_0_: the inner-potential correction. R factor: goodness of fit. Error bounds for the structural parameters obtained by EXAFS spectroscopy were estimated as CN ± 20%; R ± 1%; σ^2^ ± 20%.

**Table S3.** The sequences of siRNA.

| siRNA | Sense | Antisense |
| --- | --- | --- |
| siTRPML1-1 | CGACACAUUUGACAUUGAUTT | AUCAAUGUCAAAUGUGUCGTT |
| siTRPML1-2 | GCGGCUGGAAUUUGUCAAUTT | AUUGACAAAUUCCAGCCGCTT |
| siTRPML1-3 | GAUCACGUUUGACAACAAATT | UUUGUUGUCAAACGUGAUCTT |
